# From pre-processing to advanced dynamic modeling of pupil data

**DOI:** 10.3758/s13428-023-02098-1

**Published:** 2023-06-22

**Authors:** Lauren Fink, Jaana Simola, Alessandro Tavano, Elke Lange, Sebastian Wallot, Bruno Laeng

**Affiliations:** 1 Department of Music, Max Planck Institute for Empirical Aesthetics, Grüneburgweg 14, 60322 Frankfurt am Main, Germany; 2 Department of Psychology, Neuroscience & Behavior, McMaster University, 1280 Main St. West, Hamilton, Ontario L8S 4L8 Canada; 3 Helsinki Collegium for Advanced Studies, University of Helsinki, Helsinki, Finland; 4 Department of Education, University of Helsinki, Helsinki, Finland; 5 Department of Cognitive Neuropsychology, Max Planck Institute for Empirical Aesthetics, Frankfurt am Main, Germany; 6 Department of Literature, Max Planck Institute for Empirical Aesthetics, Frankfurt am Main, Germany; 7 Institute for Sustainability Education and Psychologyy, Leuphana University, Lüneburg, Germany; 8 Department of Psychology, University of Oslo, Oslo, Norway; 9 RITMO Centre for Interdisciplinary studies in Rhythm, Time, and Motion, University of Oslo, Oslo, Norway

**Keywords:** Correlation, Regression, Convolution, Phase coherence, Recurrence, Scale-free dynamics

## Abstract

The pupil of the eye provides a rich source of information for cognitive scientists, as it can index a variety of bodily states (e.g., arousal, fatigue) and cognitive processes (e.g., attention, decision-making). As pupillometry becomes a more accessible and popular methodology, researchers have proposed a variety of techniques for analyzing pupil data. Here, we focus on time series-based, signal-to-signal approaches that enable one to relate dynamic changes in pupil size over time with dynamic changes in a stimulus time series, continuous behavioral outcome measures, or other participants’ pupil traces. We first introduce pupillometry, its neural underpinnings, and the relation between pupil measurements and other oculomotor behaviors (e.g., blinks, saccades), to stress the importance of understanding what is being measured and what can be inferred from changes in pupillary activity. Next, we discuss possible pre-processing steps, and the contexts in which they may be necessary. Finally, we turn to signal-to-signal analytic techniques, including regression-based approaches, dynamic time-warping, phase clustering, detrended fluctuation analysis, and recurrence quantification analysis. Assumptions of these techniques, and examples of the scientific questions each can address, are outlined, with references to key papers and software packages. Additionally, we provide a detailed code tutorial that steps through the key examples and figures in this paper. Ultimately, we contend that the insights gained from pupillometry are constrained by the analysis techniques used, and that signal-to-signal approaches offer a means to generate novel scientific insights by taking into account understudied spectro-temporal relationships between the pupil signal and other signals of interest.

## Introduction

Technological advances in the last half-century, progressing from manual photography to infrared camera and eye-tracking computers, have made pupillometry an increasingly low-cost and popular methodology. The size of the pupil became the focus of interest in psychology about 50 years ago with studies on mental effort and motivational interest. The list of applications of the pupillometric method includes psychiatric and clinical studies (Rukmini et al., 2019; Kremen et al., 2019; Joyce et al., 2018; Granholm et al., 2017; Lim et al., 2016; Steinhauer & Hakerem, 1992), developmental and animal psychology research (Chatham et al., 2009; Hepach et al., 2015), neurophysiology (Gamlin et al., 2007; Reimer et al., 2016; Joshi et al., 2016), and cognitive neuroscience (Schwalm & Jubal, 2017; Urai et al., 2017).

The key processes associated with changes in pupil size are summarized in Table 1. As can be seen, the pupil is associated with a variety of states, some of which may, on their face, seem to bear no similarity to each other (e.g., fatigue and uncertainty); however, most of these states can be conceived of in relation to arousal (fatigue = low arousal; uncertainty = high arousal). Indeed, much of the interest in pupillometry stems from the proposed relationship between the pupil and the noradrenergic system of cognitive arousal. A better understanding of the neural underpinnings of changes in pupil size will make it clearer why these myriad processes are associated – still in a largely mysterious way – with small movements of the pupil and may be driven by the same system or by a few interacting systems (see “Neuralunderpinnings of pupil dynamics”).

**Table 1:** Cognitive processes associated with pupil size dynamics

Cognitive process	Key papers	Key findings
Mental effort	Kahneman and Beatty (1966); Kahnemann and Beatty (1967); Johnson (1971); Kramer et al. (2013)	Pupil size sensitive to variations in effort (greater effort, greater pupil size)
Attention (general)	Kahneman (1973); Hoeks and Levelt (1993); Iriki et al. (1996); Smallwood et al. (2011); Wierda et al. (2012)	Pupil signal indexes changes in attention over time. Pupil time course can be modeled via attentional pulses. Pupil exhibits greater spontaneous fluctuation when attention is decoupled from task
Attention (spatial)	Mathôt et al. (2013); Binda et al. (2013); Naber et al. (2013a)	When focusing attention on a visual object (even covertly), the pupil adjusts to the objects’ brightness
Uncertainty, decision-making	Friedman et al. (1973); Einhäuser et al. (2008); Jepma and Nieuwenhuis (2011); Laeng et al. (2012); de Gee et al. (2014); Urai et al. (2017); Kawaguchi et al. (2018); Colizoli et al. (2018a)	Pupil diameter increases prior to perceptual shifts and decision-making. Negative relationship between pupil size and decision confidence
Surprise, salience, orienting, prediction error	Beatty (1982a); Preuschoff et al. (2011); Wang et al. (2014); Fink et al. (2018); Alamia et al. (2019)	Pupil dilation response to surprising / alerting / salient stimuli, even when below perceptual threshold or unconscious and irrelevant to the task. Positive relationship between pupil size and prediction error
Sexual or emotional arousal	Hess and Polt (1960); Bradley et al. (2008)	Greater arousal correlated with greater pupil size. Heart rate and skin conductance also correlated with pupil activity
Response preparation, motor activity	Einhäuser et al. (2008); Reimer et al. (2014); McCloy et al. (2016)	Making a motor response (e.g., a button press) increases pupil dilation, which begins prior to the motor response. In mice, fluctuations in pupil size correlated with locomotion activity
Fatigue, task performance	Lowenstein et al. (1963); Beatty (1982a); Aston-Jones and Cohen (2005); Murphy et al. (2011); Eldar et al. (2013); McGinley et al. (2015); Knapen et al. (2016)	Performance on a task follows an inverse U-shaped function, with optimal performance at intermediate pupil sizes. Tonic pupil size decreases with time-on-task. Pupil size and behavior are correlated with changes in neural gain
Imagined arousal, effort, or brightness	Whipple et al. (1992); Laeng and Sulutvedt (2014); Sulutvedt et al. (2018); Kang and Banaji (2020)	Even during imagery or visual illusions, the pupil follows the same dilation patterns as observed during naturalistic conditions
Memory and familiarity	Võ et al. (2008); Kafkas and Montaldi (2011); Naber et al. (2013b); Papesh et al. (2012); Gomes et al. (2021)	Greater pupil size for greater familiarity; however, false alarms also produce pupil dilation (i.e., the pupil may not distinguish accurate familiarity, rather participants’ belief). Greater pupil size at encoding predicts greater retrieval success. cf. Beukema et al. (2019) and “Surprise” section above

We first briefly summarize the historical context of pupillometry in psychological research, as well as the neural underpinnings of changes in pupil size, before moving to our key concern in this article: the analysis of pupil data. We briefly outline possible data pre-processing steps, with a focus on why, how, and in what context each step might be employed; we do not attempt to define a standard but rather to increase awareness around the function of each possible pre-processing step and for which kinds of later analyses it may or may not be relevant. We then provide a sampling of pupil analysis approaches that are epoch- and/or condition-based, to situate a discussion of why one might want to employ more dynamic, signal-to-signal analysis approaches.

Though analyzing mean pupil size in a temporal window of interest has served psychology well for the last half century (and will undoubtedly continue to do so), we aim to show that a variety of powerful inferences may be possible by using more complex analysis techniques which take into account the temporal and/or spectral dynamics of the pupil signal. We are particularly focused on methods to relate the dynamic (i.e., changing over time) pupil signal to a dynamic stimulus (e.g., music, speech). Details about each analysis method and links to further reading and code implementations are provided. Additionally, we provide a code-based tutorial to recreate some of the key examples discussed in this paper. Our goal is to provide a concise and practical overview of existing methods, for those who are interested in pursuing pupillometry research but may lack the appropriate background, either in terms of the history of pupillometry or the conceptual understanding of difficult analysis techniques.

### Pupillometry

Irene Loewenfeld, in her monumental monograph on pupillometry in two volumes (1999), pointed out that there is centuries-old anecdotal and semi-scientific knowledge that the diameter of the pupil changes not only in relation to the amount of light entering the eye but also – sometimes visibly – to an individual’s internal states. The pupils were early described poetically by Joshua Sylvester (1563-1618) as “windows of the soul.” It is now common lore that dilated pupils convey the impression of someone looking both “interested” and “interesting” (explaining the cosmetic use of the herbal substance ‘belladonna’ in the Renaissance (Simms, 1967), an idea further popularized by the pioneer of pupillometry in psychology, (Hess, 1975a)).

Until the invention of infrared eye trackers, by which dynamic changes in the size of the pupil can be measured accurately, pupillary changes were simply observed with the naked eye (e.g., during neurological or ophthalmological examinations (e.g., Wilhelm et al., 1999; 2002) or by filming the eye at close range and measuring frame-by-frame the pupil diameter from the film projection (e.g., as done in the classic studies by Hess and Polt (1960, 1964) and Kahnemann and Beatty (1966; 1967). The modern infrared technology was initially developed by physiologists but was revolutionized by the development of computerized systems linked to infrared camera and specialized software for basic eye-data analysis and visualizations. Infrared light cameras also have the advantage of obtaining images in virtual darkness (for the human eye) and independently of eye colors (which vary in iris contrast on standard film).

Modern infrared eye-trackers provide raw data about pupil diameters as samples (either coded by sample frequency or by the computer clock’s time) expressed in arbitrary values (like the number of pixels of the camera) or in ‘mapped’ millimeters (after a calibration routine that also establishes head and eye distance from the camera). Nowadays, several types of eye trackers are commercially available. Broadly, they are stationary (by positioning the camera close to a computer screen) or mobile (head-mounted or integrated into glasses-like frames or within virtual reality goggles). These computerized infrared systems are capable of measuring not only how pupil size may change on average but also the dynamic movements of the pupil over time.

Within psychology, pupillometry has become the standard term, especially after Janisse’s book by the same name (Janisse, 1977). The method entered experimental and cognitive psychology due to several influential publications (Hess & Polt, 1960, 1964; Kahneman & Beatty, 1966; Beatty & Wagoner, 1978; Ahern & Beatty, 1979b). In ’Attention and Effort,’ Kahneman (1973) put forward the idea that pupillometry is a measure of attention and, specifically, of an important aspect of it: load on capacity. He proposed a psychophysiological model of ‘cognitive effort’ where pupil diameters reflect, first of all, the general physiological arousal at each moment but, more specifically, how intensively the cognitive system ‘works’ at a specific moment in time and draws on its limited resources. Subsequent physiological advances on the role of the noradrenergic system of the brain (Aston-Jones & Cohen, 2005) have provided a functional neural basis for cognitive arousal and its energizing role (tonic and phasic) on the activity of various neural systems, all concomitantly reflected in changes of the diameter of the pupil. Hence, both arousal and pupillary size are relevant variables within the current cognitive and affective neurosciences (Aston-Jones et al., 2007), with animal studies able to directly probe the activity in the noradrenergic system of the brain and its relation to pupillary changes (see e.g., Joshi & Gold, 2020 and “Neural underpinnings of pupildynamics” below).

In psychology, the appeal of pupillometry may be also due to pupillary changes being difficult to control voluntarily, unlike other oculomotor dependent measures. Control of pupil size seems only possible after extensive training (Eberhardt et al., 2021) and/or the use of indirect methods or strategies (Loewenfeld & Lowenstein, 1999). This feature of automaticity or reflexive response of the pupil to internal states seems to offer a “window into the innermost mind” (Hess, 1975b) and into mental processes that generally occur below the threshold of consciousness (Laeng et al., 2012; Fink et al., 2018). Since the initial studies within experimental psychology, pupillometry has spread into social (e.g., Goldinger et al., 2009) and developmental psychology (e.g., Hepach et al., 2015). Pupillometry is relevant in the study of low-level processes (e.g., light reflex, near response), mid-level processes (alerting and orienting), and high-level processes (executive functioning), as recently summarized by Strauch et al. (2022). Readers wishing to learn more about the basics of pupillometry are directed to the existing relevant reviews (e.g., Beatty, 1982b; Mathôt, 2018; Zekveld et al., 2018; Winn et al., 2018; Steinhauer et al., 2022; Strauch et al. 2022) or book chapters (e.g., Beatty et al. (2000); Einhäuser (2017); Laeng and Alnaes (2019)).

### Neural underpinnings of pupil dynamics

Pupil dilation and constriction are controlled by the smooth dilator and sphincter muscles of the iris, respectively. The sphincter muscle is innervated by parasympathetic axons from the Edinger–Westphal nucleus, while the dilator muscle is enervated by sympathetic axons from the superior cervical ganglion (Loewenfeld & Lowenstein, 1999; Samuels & Szabadi, 2008b, a; Szabadi, 2012). The contribution of these two different pathways can be experimentally dissociated by conducting experiments in a dark room, which reduces parasympathetic tone such that the majority of the pupil dilation response is a result of sympathetic activity (Steinhauer & Hakerem, 1992; Steinhauer et al., 2004), or by using pharmacological agents that block cholinergic or adrenergic receptors in the iris, or mydriasis eye drops (e.g., tropicamide). Such studies have confirmed, for example, that the observed pupil dilation response to an alerting stimulus can be dissociated into two components: an earlier parasympathetic one (600–900 ms) and a later sympathetic one ($\sim$1200 ms) (Steinhauer & Hakerem, 1992), and that transient decreases in parasympathetic arousal precede perceptual switches (Nakano et al., 2021) (see Steinhauer et al. (2022)’s Section 1.2.4 for additional discussion).

Neural activity in the locus coeruleus (LC) is highly correlated with changes in pupil size, in both animals and humans (Aston-Jones & Cohen, 2005; Alnæs et al., 2014), corroborating the belief that changes in pupil size reflect the functioning of the locus coeruleus–noradrenergic (LC-NA) system (Aston-Jones & Cohen, 2005; Aston-Jones et al., 1994; Minzenberg et al., 2008; Murphy et al., 2014; Nassar et al., 2012; Rajkowski, 1993). LC-NA activity affects the pupil dilation pathway, with direct stimulation of the LC resulting in a dilation of the pupil within a few hundreds of milliseconds (Joshi et al., 2016).

However, recent evidence suggests that the activity of a variety of brain regions, in addition to LC, is correlated with pupil size changes and may even be capable of driving dilation (Joshi et al., 2016; Wang et al., 2014). For example, Wang et al. (2014) show that the pupil exhibits a similar multiphasic, transient response to both visual and auditory stimuli, and assert that the intermediate layer of the superior colliculus (SCi) is likely the brain region responsible for integrating auditory and visual stimuli and interacting with the nuclei controlling pupil size. In their study, stimulation of the SCi yielded a similar pupillary response to that evoked by visual stimuli; this effect was not observed when stimulating in the superficial layers of the SC. Wang et al. (2014) offer a neuroanatomical model outlining the neural circuitry likely involved in mediating the pupillary response, which they later refine in Wang and Munoz (2015). According to their neurophysiological model, cognitively driven changes in pupil size could occur without any involvement of the LC, as could sensory-driven changes. Wang and Munoz (2015) position the mesencephalic cuneiform nucleus as the critical area receiving signals from the SC and communicating with the pathways controlling pupil dilation and constriction.

Joshi et al. (2016) also highlight some important issues with the LC-NA model. In their study of non-human primates, Joshi et al. (2016) micro-stimulated sites in the LC/subcoeruleus, inferior colliculus, and SCi. They showed that stimulation in each site caused a transient pupil dilation within 1 s (Joshi et al., 2016). They analyzed pupil vs. neural activity on multiple time scales and during spontaneous vs. evoked (tone burst) activity and showed that the LC is not necessarily the region in control. The delay until pupil dilation after LC stimulation was slow enough (500 ms) to suggest the involvement of an indirect pathway. In a recent review paper, Joshi and Gold (2020) summarize evidence suggesting that pupil size modulations can occur via three possible pathways, involving the LC, SC, or pretectal olivary nucleus (PON), respectively. The PON pathway is a direct one (i.e., there exist direct anatomical connections from the retina to the PON and back to Edinger–Westphal nucleus) and is known to be involved in pupil constriction and the pupillary light reflex; the SCi pathway is thought to be both direct and indirect, and is involved in the orienting or saliency response; the LC-NA pathway also seems to have direct and indirect connections to pupil dilation and constriction and influences pupil-linked arousal and cognition (please see Joshi & Gold, 2020 for anatomical diagrams and further details).

The neuromodulatory influences over pupil size may also be more complex than previously thought. Cholinergic (Reimer et al., 2016), dopaminergic (de Gee et al., 2014), and serotonergic (Schmid et al., 2015) activity have all been shown to correlate with changes in pupil size. However, it is possible that the activity of these three neuromodulatory systems may nonetheless be tied to LC-NA activity. Noradrenergic neurons from LC project to ACh neurons in the basal forebrain (Jones, 2004), dopaminergic nuclei are connected with the LC (Sara, 2009), and serotonergic effects on pupil size may be the result of interactions with the LC-NA system (see Larsen & Waters, 2018 for further discussion on this topic). Future studies are needed to definitively determine the complex interactions of the neuromodulatory systems and neuroanatomical pathways capable of influencing pupil size.

Summarizing across the psychological and neural underpinnings of pupillometry, readers may be left with the question of what the purpose of the pupil response is. Why is it that “higher” level processes like mental effort should be connected with the neural systems that control a light reflex, all indexed by pupil size? One way to explain the commonalities is by the underlying neurophysiological processes that are based on an activation-inhibition circuit. The LC modulates the activity of the Edinger–Westphal nucleus by inhibiting it – hence reducing the activity that leads to constrictions of the pupils – while at the same time providing excitatory signals to sympathetic circuits that directly stimulate the dilator muscles of the pupil. In other words, whenever the ascending arousal system – of which the noradrenergic LC is a key center – becomes active (e.g., because of cognitive or affective processing) the pupil dilates in proportion of the LC activation (e.g., Alnæs et al., 2014). Another way to think about commonalities is in terms of behavioral relevance. The pupil is part of an active visual system, which helps us to better explore or detect stimuli so that larger pupils provide higher sensitivity for faint stimuli or when illumination is low, whereas smaller pupils provide sharper acuity. Such an over-arching principle helps us to understand the connection between low and medium level pupillary responses, such as attentional orienting, and several scholars (e.g., Laeng & Alnaes, 2019; Mathôt, 2018) have pointed out that, even with respect to higher level processes, the nervous system, as a whole, should prime itself for an optimal response. For example, when the system is already nearer to load capacity, increased pupil size (due to load) might act as a compensatory mechanism for making sure that important changes in the environment are not missed. A possibility is that, at some evolutionary point, enlarging the pupil would enhance sensitivity to numerosity, which could confer advantages in a prey/predator situation (e.g., Castaldi et al., 2021). Of additional relevance, at least in mice, it has been shown that pupil dilation alters visual sensitivity via a switch from rod to cone-dominated visual responses – a result of the change in the amount of light hitting the retina (Franke et al., 2022). This alteration in spectral sensitivity is causally related to pupil size changes and naturally occurs during periods of increased behavioral activity, in this case locomotion, and is thought to be a behaviorally relevant mechanism to aid in predator detection (Franke et al., 2022). Further research and theorizing are, of course, required in humans. To that end, a better understanding of the relationship between pupil dynamics and other ocular motor behaviors will help in forming a more integrated view of the nervous system. Similarly, alternative analysis techniques may help to elucidate or differentiate specific pupillary functions.

### The relationship between pupillary activity and other oculomotor behaviors

As might be implied by the seemingly critical role of the superior colliculus (SC) described above, changes in pupil size have a special relationship to other oculomotor behaviors, such as saccadic movements and blinks.

#### Saccades and microsaccades

Visual processing is not uniformly distributed throughout the visual field, which makes it necessary that the eyes move to acquire visual information via foveation. Saccades are rapid, conjugate eye movements that occur about 2–3 times per second. Visual processing mostly occurs in between two saccades, when the eyes are seemingly still. However, eye movements are always present and three types of fixational eye movements have been defined: slow movement (drift), superimposed by high-frequency jitter (tremor), interrupted by high-velocity movements (microsaccades). Fixational eye movements, traditionally regarded as noise, have been demonstrated to strongly contribute to high visual acuity (see Rucci & Poletti, 2015 for a review of these concepts). Here, we focus on saccades and microsaccades, which seem to share the function of foveating regions of interest (Rucci & Poletti, 2015).

Both saccades and microsaccades are controlled by the superior colliculus and are linked to shifts in covert attention (Hafed et al., 2009). The SC – more specifically, the intermediate layers of the SC (SCi) – are thought to be an integral part of the pupil dilation response circuit (Joshi et al., 2016; Wang et al., 2014). The SCi receives input from visual, auditory, somatosensory, and fronto-parietal areas, as well as from the superficial layers of the SC (which only receive early visual input). In line with the notion that the SCi is crucial for multi-sensory integration, (Wang et al., 2017) find that pupil dilation, saccade response time, and microsaccade inhibition are correlated variables, and all exhibit greater responses in audiovisual orienting tasks, compared to solely audio or visual tasks. In earlier studies, they showed that microstimulation of the SCi (but not superficial SC layers) in monkeys led to transient pupil dilation (Wang et al., 2012, 2014; Wang & Munoz, 2014) and argue that the SCi acts as a coordinator of orienting responses, which can be measured via pupil, saccades, and microsaccades (Wang et al., 2017).

However, while saccades, microsaccades, and pupil size changes become correlated in response to a salient stimulus, it is not necessarily the case that they correlate at rest. For instance, Joshi et al. (2016) report that during stable fixation only a small proportion of the measured pupil events contained microsaccades and that those microsaccades did not occur with any consistent phase angle to the timing of pupil change events. As always, context is an important factor to consider.

The relationship between ocular motor behaviors likely changes as a function of the task at hand, tonic level of arousal, and other autonomic factors, such as the heartbeat. It has recently been shown that both microsaccades (Ohl et al., 2016) and changes in pupil size are coupled to heart rate (Wang et al., 2018). Further, Ohl et al. (2016) posit that heartbeat-evoked neural responses are capable of creating fluctuations in the oculomotor map of the SC. Such fluctuation would in turn affect the generation of saccades, microsaccades, and possibly changes in pupil size. Indeed, some have used variations in pupil size to reconstruct the heart rate rhythm and shown that pupil size variation is synchronized with very low frequency (0.0033$-$0.04 Hz), low frequency (0.04$-$0.15 Hz), and high frequency (0.15$-$0.4 Hz) cardiac rhythms (Park et al., 2018).

#### Blinks

Spontaneous blink generation has been linked to striatal do- paminergic functioning (Colzato et al., 2009; Esteban et al., 2004; Jongkees & Colzato, 2016), cf., (Sescousse et al., 2018; Dang et al., 2017), with disruptions in typical eyeblink patterning observed in clinical conditions involving timing and motor impairments (Deuschl & Goddemeier, 1998; Esteban et al., 2004; Karson et al., 1990; Nakano et al., 2011; Shultz et al., 2011; Tavano & Kotz, 2021). Blinks are thought to index endogenous attention; they increase in frequency in conjunction with an increase in Default Mode Network activity and decrease in Dorsal Attention Network activity (Nakano, 2015; Nakano et al., 2013). Blinks are known to occur at structurally salient breaks during reading and speech (Cummins, 2012; Hall, 1945) and to be indicators of cognitive event chunking, as well as cognitive load (Siegle et al., 2008; Stern et al., 1984). Blinks increase whilst speaking, in conjunction with increased facial motor activity (Orchard & Stern, 1991) and are likely to become synchronized between speakers (Nakano & Kitazawa, 2010).

With regard to pupil size, Siegle et al. (2008) show that the proportion of blinks at any given moment in time (averaged across trials, per sample) closely mirrors the pupillary response, during a cognitive load task. Their data suggest that an increase in blink activity precedes an increase in pupil size and that instances of greater blink activity tend to occur when the pupil signal is stationary in terms of acceleration (i.e., not accelerating or decelerating, in other words, when the second derivative of pupil size nears 0). A sustained increase in proportion of blinks was observed following pupil dilation. Interestingly, Siegle et al. (2008) find that blinks at the beginning of a trial are correlated with pupil dilation at a later stage (4–10 s later in a Stroop task), suggesting that the cognitive load indexed by pupil dilation is proportional to the blink response at initiation of the cognitive event. Though blinks and pupil size changes were correlated, Siegle et al. (2008) suggest that they provide unique information, with blinks being more sensitive to event onsets and offsets, and pupil dilation more sensitive to on-going processing.

Such a finding of blinks correlating with the pupil signal, even seconds later, is in line with the more recent work of Klingner et al. (2011) and Knapen et al. (2016). Knapen et al. (2016) show that blinks explain approximately 40% of the variance in pupil data, and show pupil effects lasting approximately 5 s after a blink. In their data, a blink causes a rapid decrease in pupil size, followed by a seconds-long increase. To correct for this, they model the pupillary response to a blink with a double gamma function, find instances of blinks in the pupil data, and deconvolve the blink-related pupil response from the data. Klingner et al. (2011) also determined that blinks affect the timing and magnitude of the pupil signal, and show differences in the pupillary response to the blink which depend on the duration of the blink. To counteract the possibility that blinks were systematically biasing their pupil data, they grouped blinks by duration and calculated an average pupillary response for each blink duration, which they then subtracted from the relevant portion of their pupil data. This average response typically consisted of a brief dilation, followed by constriction, followed by an approximately 2-s return to baseline pupil size, though the timing and magnitude of the changes were a function of blink duration. Interestingly, Klingner et al. (2011) report that their pupil results remained the same whether using their blink correction method as described above or only using standard blink interpolation. Similarly, Zénon (2017) reports a significant negative relationship between pupil dilation and blinks (i.e., pupil constriction after a blink) but shows that, even when accounting for such correlations in a statistical model, the main results related to pupil dilation and arousing images are not affected. Quirins et al. (2018) also find little difference in their results depending on whether they reject blink trials or interpolate pupil data during blinks.

In sum, these studies speak to the importance of at least checking the relationship between blinks and pupil dilation and quite possibly correcting for it using a subtractive or regressive technique. In particular, for tasks in which blink activity is highly correlated with stimulus events in one condition but not another, not correcting for blinks may significantly bias the results. Nonetheless, a balance between theoretical considerations and pragmatic ones must be struck. We discuss such issues further in the pre-processing considerations below.

## Pre-processing pupil data

We outlined the neural underpinnings of changes in pupil dynamics, as well as the relationship between changes in pupil size and other oculomotor behaviors to stress the importance of understanding what exactly is being measured when recording pupil size and what can and cannot be inferred from changes in pupillary activity. However, equally important is to note that the insights one can gain from pupillometry are constrained by the analysis techniques one uses. Before arriving at analysis approaches, a consideration of pre-processing steps is necessary. However, we emphasize that the pre-processing steps one chooses should be dependent on one’s planned analyses. For example, many time-series based techniques discussed below require the pupil signal to be evenly sampled and contiguous. This means that pupil data during blinks, saccades, or other moments of data loss should be imputed or interpolated. Further, all pupil data for all participants should be at the same sampling rate and ideally contain the same number of samples. However, depending on the eventual statistical model one wishes to use, some pre-processing steps might become unnecessary: for example, gaze position (van Rij et al., 2019) or blinks (Zénon, 2017) can be entered into statistical models as co-variates, rather than corrected for in the pupil signal; interpolation and filtering should be avoided when using GAMMs, as they increase autocorrelation in model residuals (van Rij et al., 2019; Wood, 2020). Below, we outline possible components of a possible pre-processing pipeline – not a required list. We provide basic details about each possible pre-processing operation and discuss considerations with respect to eventual analysis techniques. Regardless of the pre-processing steps implemented, we cannot stress enough the importance of visualizing data and checking for outlying samples, spikes, etc. In the accompanying code tutorial, readers can explore pupil data for different participants’ and think through potential issues and sources of noise (see code tutorial section *Explore basics of time series)*.

### Discarding trials in which too many pupil data points are missing or noisy

Missing data occurs when the pupil size goes to zero, resulting either from a blink or from the eye-tracker’s loss of the pupil. Noisy or problematic data are typically registered via a flag output by the eye-tracker for each pupil sample indicating whether it is valid or invalid, or, alternatively, a continuous measure of tracking quality or confidence (N.B. eye-trackers handle this procedure differently, depending on the manufacturer’s choice and scientific tradition). Missing and invalid pupil data should be set to “not a number” (NaN) for future pre-processing (i.e., interpolation). One way of automating such a process would be to set a threshold-based rule, like, “if greater than *x*% of the pupil data are missing, the run is discarded.” Note that there is no decisive rule for percent missing data permissible; note also, that, if baseline periods are being used, missing data may need to be evaluated separately for baselines vs. trials (see also “Baseline correctingpupil data”).

### Removing improbable data

Mathôt et al. (2018) suggest setting a cutoff threshold (based on visualization of pupil size distributions, not predetermined rules like 2 standard deviations) and removing outlying data points. Perhaps more broadly applicable, Kret and Sjak-Shie (2019) suggest removing outlying pupil data points that 1) contain unrealistic changes in dilation speed or 2) are isolated from surrounding data (e.g., a sparse data point that my occur in the midst of a blink when the eye-tracker erroneously measures the pupil for a few samples or tracks other elements of the face, like eye lashes, especially if the participant has applied mascara). The authors provide equations and code for enacting these cleaning procedures. Please note that though the word “removing” is used, we do not literally mean removing those data points and shortening the signal, we mean setting problematic data points to “empty” or NaN. These empty data points can later be interpolated.

### Interpolating missing data

Interpolation involves fitting a line, or quadratic function, to fill in missing data between existing data points. If not due to poor recording quality or participant movement, there will always be brief periods of loss, or extreme values, in the pupil signal due to blinks. Typically, these periods, plus some padding on both sides (usually 50–200 ms), are set to NaN (see section above), then interpolated. Whether to use linear or cubic spline interpolation is a matter of personal preference, as there seems to be no consensus in the extant literature. While fitting a quadratic function (cubic spline interpolation) may more closely mimic the natural fluctuations of the pupil, it can also lead to a wider variety of introduced artifacts as compared to the fitting of a simple line.

Note, however, an alternative to interpolation would be to leave all missing samples as NaN, especially if one only needs to compute the average pupil dilation response in an epoch, or if one plans to use GAMMs. On the contrary, many signal processing techniques (e.g., a fast Fourier transform) require continuous data and cannot handle a time series with empty samples as input. Therefore, in such cases, interpolation becomes a necessity. Readers are thus reminded to think carefully about their particular use case before applying such corrections, and to visualize any signal transformations they employ, such as interpolation, to be sure that no artefacts have been introduced in the process.

### Modeling the pupillary response to blinks and saccades using regression

As foreshadowed by the discussion of the relationship between pupillary activity and other oculomotor behaviors above (“The relationship between pupillary activity and otheroculomotor behaviors”), it is important to control for a variety of other oculomotor parameters when analyzing changes in pupil size. One solution is removal and/or interpolation (outlined above). Another solution is Knapen et al. (2016)’s method to model the pupillary response to both blinks and (micro)saccades and deconvolve those stereotyped responses from the pupil data (for more details about the basics of convolution see “Pupillary response function”). Because they show that the effect of blinks and saccades on pupil size lasts approximately 5 s, a method like interpolation will not remove the long-term artifact caused by these oculomotor behaviors, making the deconvolution method a possibly necessary step (notice that interpolation should still be conducted beforehand). For those interested in implementing Knapen et al. (2016)’s finite-impulse-response fitting method, Python code and tutorials are provided alongside the original paper.

As Knapen et al. (2016)’s artifact removal method has only recently been suggested, it is not yet widely adopted. One potential issue is that one needs enough observations of blink and saccade-related pupil activity to estimate a valid deconvolution kernel for those events (i.e., to build a model of saccade and blink-related pupil activity, respectively). Such models will be difficult to estimate if participants rarely blink, for example. One may need to employ specific experimental design choices to ensure enough blinks for a valid model (e.g., allowing participants to blink freely, having a long baseline period in which blinks are sure to occur, etc.). Pragmatically speaking, if blinks or saccades rarely occur, they are probably negligible. Even though they may add measurable noise to the pupil signal, such noise may make no significant difference in terms of statistical results. Nonetheless, the relative frequency and magnitude of blinks and saccades should be assessed. The most important check is that blinks and saccades do not occur in some experimental condition with greater frequency and magnitude vs. another, thus possibly biasing pupil results and interpretations. If significant differences exist for blinks or saccades in certain conditions, those should be reported and the researcher should be careful to control for such confounds in the pupil data.

### Filtering

A high-pass filter can be used to remove large-scale (low frequency) drift in pupil data, while a low-pass filter can be used to remove physiologically irrelevant high frequency noise in the data. However, with either high- or low-pass filtering, it is important to be sure that the filtering functions being used do not affect the phase of the pupil signal or create ringing artifacts, which might later appear as activity of interest (see de Cheveigné & Nelken, 2019 for a discussion of this issue and filtering advice). Similarly, this artifactually introduced autocorrelation in the signal can be a problem for some statistical modeling approaches one might later wish to use (e.g., GAMMs; see van Rij et al., 2019). It is also important to note that low frequency information in the pupil signal might actually be of interest, since it may signal changes in tonic activity in the LC-NA system that is meaningful in terms of cognitive processing. In this case, very low or no high-pass filtering should be employed (e.g., for infraslow activity see Blasiak et al. (2013); Okun et al. (2019); for time-on-task effects, see “Accounting for time-on-task”; or for detrended fluctuation analysis see “Detrended fluctuation analysis (DFA)”). Additional examples and discussion can also be found in accompanying code tutorial section *Filtering*.

### Gaze correcting pupil data

When gaze changes occur during a task (e.g., during free-viewing or reading tasks), it is of critical importance to correct for the pupil foreshortening error (Hayes & Petrov, 2016; Gagl et al., 2011; Brisson et al., 2013) – that is, when the pupil falsely appears to have changed size due to the now different angle of the pupil to the eye-tracking camera, as a function of gaze position change. The correction technique of Hayes and Petrov (2016) is fairly straightforward but requires taking measurements of distances from the eye-tracking camera to the eye, to the screen, etc. to be used in calculating an appropriate model. Though such measurements could be easily computed in most cases, they might not be possible if the data are being accessed in an open-source context that has not documented such information. In the event that these measurements are unknown or participants are constantly presented with a fixation cross and nothing else on the screen (e.g., a purely auditory task), an alternative to gaze correction would be to remove any periods in which the eye is greater than a few degrees away from the center fixation cross (Korn & Bach, 2016). If the task only involves changes in horizontal gaze position (e.g., during text reading), then the synthetic correction function of Gagl et al. (2011) can be applied. Alternatively still, rather than correcting pupil size in the pre-processing stage, one can include *x* and *y* gaze position as regressors in a later statistical model (see e.g., van Rij et al. (2019), who include *x* and *y* gaze as nonlinear interaction terms in a generalized additive mixed model). Similarly, based on gaze position, Madsen et al. (2021) regressed out both local and global luminance from every subject’s pupil data while watching a video. The global luminance was the luminance of the entire frame, while the local luminance was a small, defined radius around the point of gaze. Note, however, that in typical cognitive psychology pupillometry experiments, the general recommendation is for eye position to remain constant between conditions (please see Mathôt & Vilotijević, 2022 for detailed discussion of relevant experimental design principals).

### Normalizing pupil data

To compare variance in the pupillary time course related to the task at hand, normalizing pupil can be useful. Several studies normalized their data in some way – for example, by calculating percent change from mean pupil size over the course of a trial (e.g., Lavín et al., 2014), by *z*-scoring the pupil data for each trial (e.g., Colizoli et al., 2018a; Kawaguchi et al., 2018; Fink et al., 2018; Wainstein et al., 2020), or by using dynamic range normalization (e.g., Piquado et al., 2010 employed a pre-test to ascertain differences in pupil response ranges between younger and older adults and correct trial data based on these individual ranges). Perhaps the most critical aspect of normalization is to clearly report the equation used so that others can easily replicate results or understand how results might diverge based on different normalization choices. To provide a few concrete examples: Fink et al. (2018) report the following equation, normalizing based on the mean and standard deviation of the trial:

1 $$\begin{aligned} x^{\prime } = \frac{x - \bar{x}}{S(x)} \end{aligned}$$

while Piquado et al. (2010) report the following equation, normalizing based on the minimum and maximum range of the pupil:

2 $$\begin{aligned} x^{\prime } = \frac{x - x_{\min }}{x_{\max } - x_{\min }} * 100 \end{aligned}$$

In deciding about data normalization, one should consider what kind of variability is relevant for the research question at hand, and operate accordingly. Again, later statistical modeling approaches that include random effects for individuals may preclude the need to normalize data.

By definition, a normalization procedure will convert the pupil data from the raw measured units to arbitrary or standardized ones. While such a transformation can have advantages for cross-participant or group comparisons, it also has some downsides. For example, the true pupil diameter value in millimeters may provide additional insights as to which type of process underlies pupillary change. Steps up or down of light intensity can change the pupil with constrictions as small as one third of its diameter or dilations that are twice as large as the diameter of the resting state. Such pupillary responses to light increments or decrements are very dramatic, compared to pupillary change due to psychological factors (like mental work or emotional states), best observed when luminance is kept constant. Psychological changes are rarely greater than 0.5 mm^3^ or approximately 15 to $20\%$ increments from rest. Moreover, given that pupil size can range between 2 and 8 mm (Watson & Yellott, 2012) and that pupil changes driven purely by sensory information (e.g., luminance or nearness) are greater than psychosensory responses makes meaningful checking the true values in millimeters (if available), since these may be an important data quality check (Mathôt, 2018). Pupils being part of human anatomy, there is an obvious advantage in expressing pupil size according to real-world dimensions, as is recommended by Steinhauer et al. (2022). However, though some eye-trackers output pupil size in millimeters, others output pupil size in arbitrary units. In the case of arbitrary units, some algorithms to convert to mm exist, if particular parameters are known (e.g., distance to the screen; see Hayes & Petrov, 2016, Fig. 4), otherwise, normalizing can offer the possibility to put pupil data recorded in arbitrary units onto the same scale across participants.

### Baseline correcting pupil data

While normalization re-scales a signal based on measured or statistical constants, baseline-correction refers to altering the pupil signal based on measurements taken during a baseline period. Such correction does not necessarily change the unit of pupil measurement (i.e., it can still be in millimeters), but it does make the reported pupil measure relative (to the baseline). The assumption is that, by taking the mean or median of the pupil size during the pre-stimulus period and subtracting (or dividing) it from the stimulation period, aspects of the pupil signal unrelated to the stimulus are removed. Such “aspects” might be person-specific (e.g., general arousal level) and/or stimulus-specific (e.g., luminance). However, Mathôt et al. (2018) show through a series of simulations that baseline correction can create large distortions in the measured pupil data (particularly if a blink occurred during the baseline period) and bias statistical results. Ultimately, they suggest using a subtractive, rather than divisive, baseline correction, as it is less susceptible to artifact. They also provide suggestions for visually inspecting baseline-corrected pupil data to check for artifacts (e.g., rapid changes in pupil size occurring in less than 200 ms following the baseline period are suspect). Similarly, Laeng and Alnaes (2019) suggest a subtractive method and advise against percentage-based corrections, in line with the first generation of researchers in pupillometry (Beatty, 1977). See also Reilly et al. (2019) for further discussion of baseline procedures and the need for standard procedures.

An alternative to baseline-correcting the pupil data of interest, is to include baseline pupil size as a regressor in a final statistical model (van Rij et al., 2019). This approach circumvents the possible issues noted above and is an elegant means to account for a variety of possible baseline effects. For example, Widmann and colleagues illustrate how such an approach unites divergent findings related to the effect of baseline pupil diameter and luminance levels on subsequent pupil diameter changes (Widmann et al., 2022). Combined with a factor analysis separating the pupil trace into parasympathetic and sympathetic components, they show that baseline pupil size has a negative linear relationship with parasympathetically mediated pupil size changes, while the sympathetic component exhibits an inverted U-shaped function. They also suggest that, given the effect of luminance level on the possible range of evoked pupil sizes, pupil data recorded at different luminance levels cannot be directly compared and should always be reported.

**Fig. 1:**
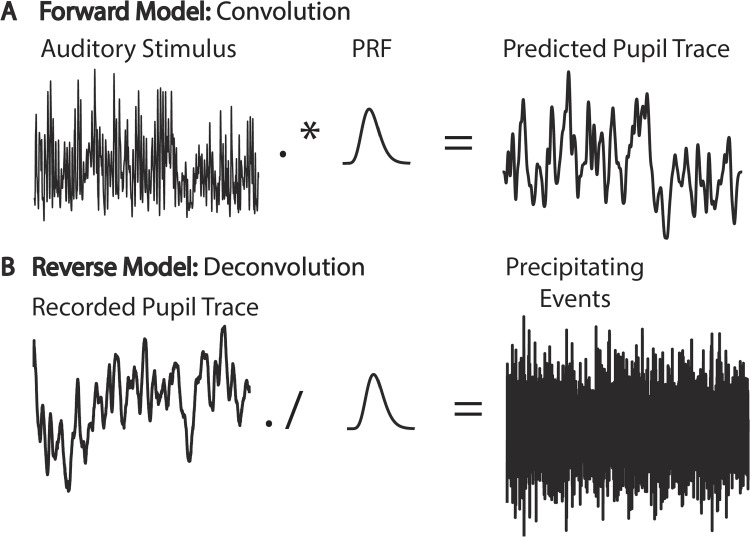
Example of using convolution (**A**) or deconvolution (**B**) to predict the recorded pupil trace or precipitating events, respectively. Note that one could use the canonical pupillary response (PRF) for each participant or estimate the PRF individually for each participant. Alternatively, estimating the PRF could be the goal of analysis in its own right (see “Temporal response function” below)

### Accounting for temporal lag

One possible limitation of pupillometry is the lag between external and/or cognitive events and the subsequent change in pupil dynamics. Such lag is still less than the lag in blood-oxygen level dependent (BOLD) signal in functional magnetic resonance imaging (fMRI), but is considerably larger than the lag of electroencephalographic (EEG) or magnetoencephalographic (MEG) signals. While such a lag should not deter one from conducting pupillometry studies, it should be carefully considered in experimental design (e.g., making sure there is enough time between the presentation of any two successive events for the pupil to return to baseline) and/or analyses (e.g., correcting pupillary responses which may have summed in time due to events occurring rapidly; see e.g., Wierda et al. (2012) and “Pupillary responsefunction”). To date, a few main approaches exist for handling lag in pupil analyses: (1) using convolution or deconvolution with a pupillary response function (PRF), (2) calculating the first derivative of the pupil signal, or (3) separately analyzing a fast and slow pupillary component. We discuss all three approaches in turn.

#### Pupillary response function

Given the various possible top-down and bottom-up influences on changes in pupil size, it is difficult to ascertain which external or cognitive events drive pupillary changes, and at what time lag. To address this issue, Hoeks and Levelt (1993) asked participants to listen to auditory tones and respond with a button press. They fit the averaged pupillary responses of participants with an Erlang gamma function. The function was estimated to have parameters $m=1$ (linear exponent), $n=10.1 +/- 4.1$ (numbers of steps in the signaling cascade) and $t_{\max } = 930 ms +/- 190 ms$ (latency of maximum pupil response). Such a function can be used to model how the pupil will respond, given some input stimulus. However, with only eight participants, during one type of task, the parameters of this pupillary response function (PRF) remain to be more widely studied in different contexts and with a larger number of participants.

More recent reports have noted that, when no motor response is required, the maximum pupil response latency is around 500 ms, and that, when a motor response is required, two peaks are present in the pupil signal, the first around 750 ms and the second, bigger peak around 1400 ms after tone onset (McCloy et al., 2016). Still others continue to refine PRF models by adding free parameters into the response function (Fan & Yao, 2010) or disregarding the biophysical reality and finding the best fitting model (Korn & Bach, 2016). Additionally, recent work shows that the time to maximum pupil dilation varies across participants but is consistent within participant, suggesting the need to fit a PRF separately for each participant (Denison et al., 2020), rather than using one canonical model for all participants.

The advantage of using a PRF is that it allows one to either forward (convolution) or reverse (deconvolution) model the predicted pupil time series or the correlated cognitive or stimulus events, respectively (see Fig. 1). The (de)convolution technique has been used in a variety of studies to show that the pupil reflects fluctuations in attention and decision-making at a fine temporal resolution; see for example: Wierda et al. (2012); de Gee et al. (2014); Kang and Wheatley (2015); Korn and Bach (2016); Korn et al. (2017); Fink et al. (2018); Denison et al. (2020). Generally defined, convolution is the integral of the product of two functions – in our case, our two times series of interest, with one reversed and shifted along the length of the other. It could also be thought of as the moving dot product calculated at each moment in time when one signal is reversed and shifted in time along the other. Still also, convolution can be thought of as a type of filter or weighting function. For example, in Fig. 1A, we see the amplitude of an acoustic signal. When we convolve that signal with our PRF, or “kernel,” we see that the output signal is now much lower in frequency content (i.e., high frequencies have been removed and the input signal is now weighted by the response properties of the pupil; in other words, in the temporal range of the pupil). To help readers gain a deeper understanding of (de)convolution, we provide an interactive demo in the accompanying code tutorial; see code sections *Convolution*, *Building intuition about how convolution works*, *Deconvolution*, and *Predicting pupil data*.

Alternative to using pure convolution, one could do the same type of analysis by optimizing a fit between the two signals, using regression-based techniques. Please see “Temporal response function” for more details about such an approach and for more information about using the pupillary response function as a dependent measure, rather than as a means to account for lag between pupil activity and the signal of interest, as explained above.

#### Pupillary difference signals

Depending on the research question, the number of pupillary changes, or the time points of change, may indeed be more interesting than evoked pupil size. For instance, in the simulated data plotted in Fig. 3C, The first and second traces show opposite polarity of pupil size (i.e., when one increases in size, the other decreases); however analyzing the derivative of both signals would show similar instances of pupillary change. Further, analyzing the derivative of pupil size allows one to examine instances of pupillary change which occur on a faster time scale. In relation to preceding events, de Gee et al. (2020) show that, in humans, the first derivative of pupil change can be observed as early as about 240 ms after stimulus onset, bringing the pupil time series onto a much faster timescale, potentially more suitable for certain types of analyses or research questions.

Beyond increasing the temporal resolution of the pupil signal, pupil derivative metrics may be interesting dependent measures in their own right, for instance in classifying clinical conditions (Fotiou et al., 2009), predicting lapses in task performance (van den Brink et al., 2016), or studying attention to auditory sequences (Milne et al., 2021). One could also count the number of changes in pupil size between conditions as a dependent measure, as has been done in both macaque (Joshi et al., 2016) and human studies (Jagiello et al., 2019; Schneider et al., 2016). Note that most of the analysis techniques discussed below in “Analysis techniques” can be conducted on the standard pupil signal or its derivative(s).

#### Pupillary components

Because the pupil is driven by both parasympathetic and sympathetic activity, another approach to understanding the temporal lag or dynamics in the system is to separate the pupil signal into different components, typically using principal components analysis (PCA; e.g., Steinhauer & Hakerem, 1992; Steinhauer et al., 2004). Such an approach has been used, for example, by Widmann et al. (2018) to show that emotionally arousing music acts on pupil dilation specifically through the sympathetic branch. In addition to segregating by sympathetic and parasympathetic, one could also separate the pupil signal into components thought to be driven by cognitive events, such as an early attentional orienting or sensory component vs. a later executive control one (see e.g., Geva et al., 2013; Geng et al., 2015). Note that PCA is typically used on the pupil dilation response over a somewhat short time window (e.g.,  3 s), and to date has not been used over longer time scales. Nonetheless, we discuss it here as one might still wish to employ some of the time series methods discussed below on these short component traces, or to attempt application of PCA to pupil time series of longer duration.

### Accounting for time-on-task

Prolonged task performance results in changes in tonic pupil diameter (i.e., time-on-task effects). For example, van den Brink et al. (2016) showed that time-on-task can impose relationships between pupil diameter and task performance that obscure the more nuanced effects of task on pupil dilation. Thus, in addition to revealing interesting phenomena, such as lapses in attention (Kristjansson et al., 2009) or changes in pupil size decrements depending on emotional content of auditory text excerpts (Kaakinen & Simola, 2020), it may be important to control for time-on-task effects in pupil diameter analysis over long time scales.

One way to take into account such effects is to apply a sliding window to the behavioral performance (e.g., accuracy or response times) and pupil data and to extract the average performance as well as pupil diameter and/or velocity (i.e., the first order temporal derivative) within each window. To examine whether the pupillary signal shows time-on-task effects, van den Brink et al. (2016) fitted a straight line to the pupil time series obtained by the moving average and used the slope of the fitted line as an index of linear trend over time. The distribution of slopes across task blocks for each participant was then compared to zero using a *t* test. Relationships between the time series of pupillary and performance measures can be examined by comparing these measures with multiple regression (see e.g., van den Brink et al., 2016). Including quadratic regressors in statistical models can reveal non-linear relationships between variables, such as the typical Yerkes–Dodson (i.e., the inverted U-shaped) relationship between pupil dilation and task performance, which is compatible with the adaptive gain theory of LC-NE function (Aston-Jones & Cohen, 2005). Such effects may be obscured if the time-on-task effects are not statistically partialled out.

**Table 2:** Signal-to-signal analysis techniques and the general question each can address

Method	Question
Correlation	To what degree do the signals change together?
Cross-Correlation	To what degree do the signals change together, when allowing for different temporal lags between them?
Inter/Intra subject correlation	To what degree do the pupil traces across participants, or within a single participants’ repeated exposure to the same stimulus, change together?
Reverse correlation	Which events lead to moments of change in pupillary activity?
Regression	How well can we predict one signal given another?
Dynamic time warping	How (dis)similar are the signals with respect to changes in amplitude, when allowing for warping in time?
Inter-site phase clustering	How well are the signals aligned in phase, at specific frequencies, irrespective of power?
Magnitude squared coherence	How well are the signals aligned in phase, at specific frequencies, when accounting for power?
Detrended cross-correlation	Do the signals exhibit similar scale-free dynamics?
Cross-recurrence quantification	Do subsections of the signals repeat in a coupled way?

Related to time-on-task, one may also wish to consider the sleepiness of participants. For example, Lüdtke et al. (1998) analyzed slow (0.0$-$0.8 Hz) pupillary oscillations as indices of participants’ fatigue. They detected slow waves by applying a fast Fourier transformation for consecutive segments of 82 s over the entire 11-min recording and plotted the power spectrum estimate for each data segment. Slow oscillations (fatigue waves) were more prominent for participants who scored high on self-rated sleepiness. They used a pupillary unrest index (PUI: cumulative changes in pupil diameter based on mean values of consecutive data sequences) to further characterize the differences between alert and sleepy participants. The median power and PUI scores were both higher in the sleepy as compared to the alert participants. Both slow oscillations reflecting fatigue and changes in pupil diameter over time-on-task thus increased when participants were sleepy. Similar observations were made in a seminal paper by Lowenstein et al. (1963). Note that the PUI may also be an interesting dependent measure in its own right, depending on the research question (see e.g., Schumann et al., 2020) and that that these low frequency oscillations have alternatively been referred to as hippus (Bouma & Baghuis, 1971) or fatigue waves (Lowenstein et al., 1963). These < 0.15 Hz oscillations are thought to be mediated mostly by parasympathetic activity, though Schumann et al. (2020) also show a relation with sympathetic measures, namely, the amplitude of pupillary responses, vagal heart rate variability, and spontaneous skin conductance fluctuations.

While one solution to account for time on task would be including regressors in statistical models, other solutions are also available. van den Brink et al. (2016) found that the derivative of pupil diameter (see “Pupillary differencesignals”) was robust to time-on-task effects, suggesting that this measure offers a potential marker of attentional performance that does not require correcting for time on task. Additionally, working with shorter (e.g., 1 s) epochs and *z*-scoring them accounts for time-on-task effects (see e.g., Madore et al., 2020). Another alternative is to restrict the analyses to pupillary responses from a subset of trials that are not affected by the time on task (see e.g., Aminihajibashi et al., 2020), or to use a high-pass filter to correct pupil drift over time (see “Filtering”). Yet another approach is to think about time-on-task effects as a special case of temporal dependency in the signal; in this case, statistical models that account for autocorrelated errors can be employed (see “Single trialmodels” and van Rij et al. (2019)).

## Analysis techniques

Whether analyzing the raw pupil trace, pupil derivative, pupil components, or (de)convolved pupil signal, the eventual goal is to characterize similarities or differences between pupil responses in different conditions, within / between participants, with respect to a given stimulus, or with respect to predicted pupil data (see Fig. 3 for examples). To date, most pupillometry papers have compared mean pupil size or the pupil dilation response across different epoched conditions of interest. This section first outlines those traditional methods based on means, before moving into ways to analyze single-trial pupil signals in both the time and frequency domains, in linear and non-linear ways. While the overall focus and interest of this paper is on signal-to-signal analysis approaches (e.g., comparing the continuous pupil signal to a continuous speech or music signal; see Fig. 3), it is critical to understand epoch-based approaches when considering whether and when to use alternative, continuous, signal-to-signal ones. Additionally, with an appropriate experimental design and planned statistical model, some signal-to-signal measures may be used within epoch-based frameworks.

Table 2 provides a summary of each of the signal-to-signal analysis techniques we discuss below and the type of question they can help to answer. In the subsection for each technique, we aim to provide (1) a conceptual understanding of the mathematical concept, (2) its application in pupillometry, and (3) references to key papers, tutorials, or code toolboxes to learn more about the technique. All code required to recreate every figure in the paper and to step through the analysis techniques with the provided toy data set, is available on GitHub and on Code Ocean.^1^

### A brief review of epoch-based approaches

The first and still widely used method for analyzing the pupil diameter (see Laeng & Alnaes, 2019 for a review) either disregards pupil data as time series or approximates it by dividing the pupil response into epochs or bins, typically based on an equal number of samples (e.g., Bianco et al., 2019; Bochynska et al., 2021; Zavagno et al., 2017). However, we wish to note that time is never really “ignored;” rather, the researcher makes the implicit assumption that the window over which they have averaged is the only relevant temporal scale of interest, thereby discarding experiment-wise changes in response patterns.

Many classical and influential studies used a statistical approach which did not take pupillary changes over time into account, although they also often presented graphs of the pupil waveform as an illustration (e.g., Kahneman & Beatty, 1966; Ahern & Beatty, 1979a), relying on the readers’ ability to perform “eyeball statistics” (i.e., viewing that some portions of the waveform belonging to different conditions or groups of participants were visibly above or below one another). In fact, some of the seminal studies by Hess and Polt (1960, 1964), which introduced the method of pupillometry into psychology, did not analyze the pupil with formal, inferential, statistics but simply showed average data in either a table or a bar graph (without any metric of error).

**Fig. 2:**
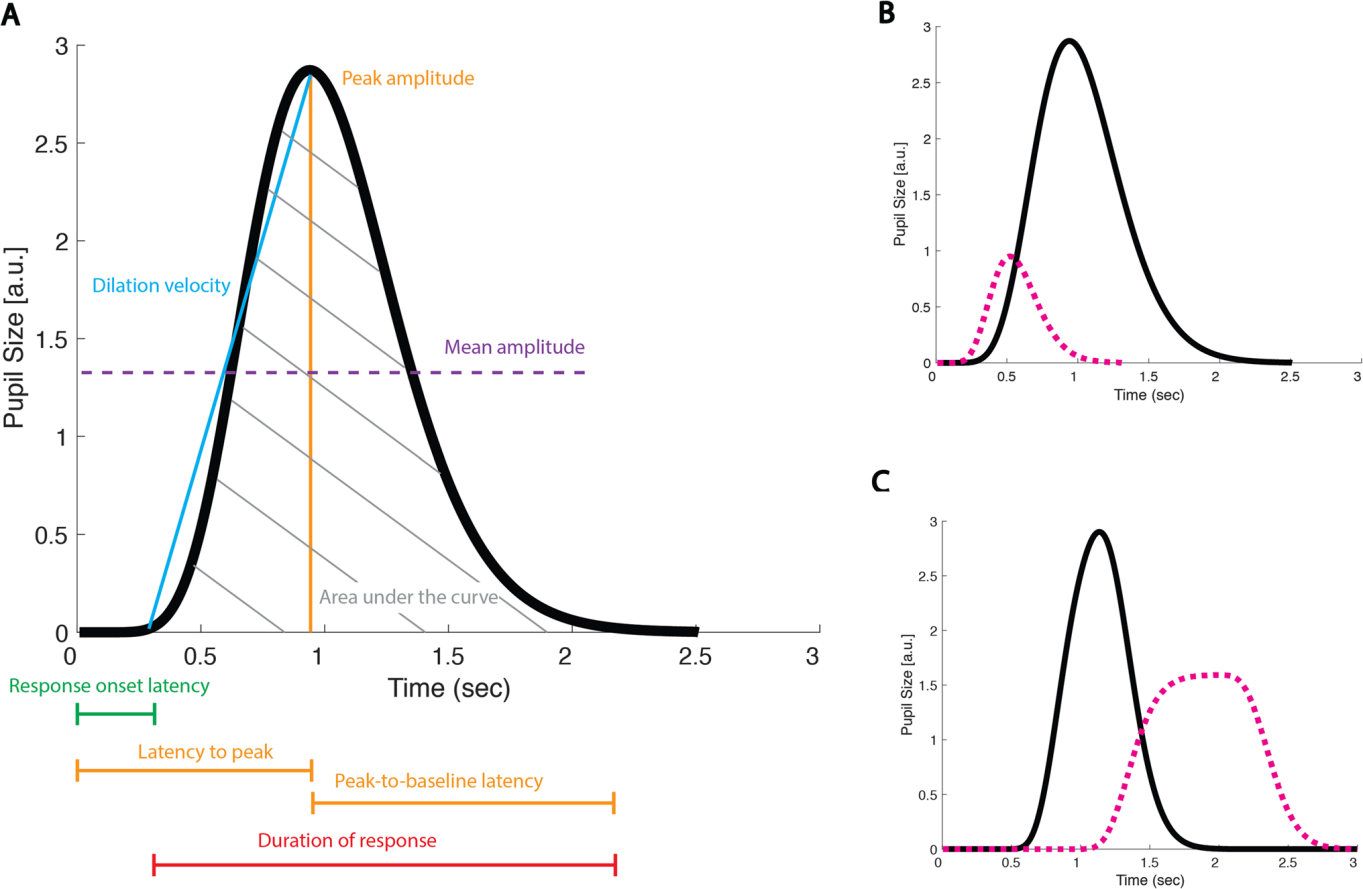
Examples of possible metrics of interest within a pre-defined epoch (**A**). Panel **B** shows two simulated pupil traces, corresponding to two hypothetical conditions of interest (*solid black line* vs. *pink line*). These two traces represent an example of when the pupil dilation response differs in mean and peak amplitude, duration of response, area under the curve, etc., but not dilation velocity. Panel **C**, on the other hand, shows example pupil dilation responses with the same mean amplitude but different response onset latencies, dilation velocities, and sustained dilations. See “A brief review of epoch-based approaches” for more details. All data are simulated from a canonical pupillary response function; see accompanying code tutorial section *Fig. 2*

Note that, though most previous studies solely analyzed mean pupil size within an epoched window (sometimes referred to as a task-evoked pupillary response, or TEPR), recent studies have turned to a variety of new epoched measures, such as maximum evoked dilation, latency until maximum dilation, dilation velocity, sustained amplitude, delay until return to baseline, or area under the curve of the dilation response (see e.g., Wang et al., 2014). Visualizations of some of these metrics are provided in Fig. 2, panel A. Panels B and C highlight cases where specific measures differ. An important point of interest to highlight is in Panel C, where taking the mean pupil size in the 3-s epoch would yield the same result for the two pupil traces (solid black and dotted pink), perhaps leading a researcher to conclude that there are no significant differences between the two conditions that correspond to those two traces. However, visualization of the pupil waveform clearly shows some potentially important differences with respect to response onset latency, peak dilation, dilation velocity, etc. We, therefore, urge researchers to visualize their pupil waveforms, rather than blindly taking means within epochs. Such visualization is also important for considering the appropriate epoch duration to choose (Steinhauer et al., 2022).

While these other metrics can clearly provide alternative insights, compared to means alone, they also present some new challenges. For example, how to define peak pupil dilation. In the black traces in each panel in Fig. 2 the peak pupil amplitude is quite obvious; however, what about the pink trace in Panel C – when exactly does the peak occur? Defining the peak also influences other possible metrics of interest, such as latency to peak or peak to baseline latency, or a metric not pictured here – referred to as peak-to-peak amplitude – which would be relevant if the pupil exhibited a positive peak, followed by a negative one. Thus, defining the peak is an important problem. Looking for the maximum value of pupil size within the epoch is, of course, the easiest way to define the peak amplitude; however such an approach can be susceptible to artifacts. An alternative might be taking a mean within the window of time between two changes in slope (see e.g., Reilly et al., 2019). However, it is also important to keep in mind that averaging waveforms can result in distorted peaks and latencies. Thus, finding peaks on the single trial level then averaging, or constructing a statistical model with single trial peak amplitudes included (see “Single trial models”), may be preferable to finding the peak of an averaged waveform. In general, pupil dilation responses can be conceived of analogously to event-related potentials (ERPs) in EEG analysis. In the ERP literature, the possible pitfalls of making assumptions from averaged waveforms (actually composed of different underlying component waveforms) and analyzing waveform peaks have both been discussed extensively. Please see Luck (2014) for thorough explanations and advice regarding best practices.

If epoched analyses, with statistical inferences, are the sole analysis aims of the reader, many recently developed software tools will work off-the-shelf. For example, CHAP (Hershman et al., 2019), written in MATLAB, provides an easy-to-use and standardized starting point. CHAP can parse input files from a variety of different eye-tracking systems and can deal with basic pre-processing steps (outlying samples, interpolation during blinks, exclusion of outlying trials, and exclusion of outlying participants). In a graphical user interface (GUI), the user can define preferred parameters for exclusion and subsequently define the trial and group level variables that are relevant for analyses. CHAP will provide epoch-based statistics and plots, with respect to the entire epoch or to changes over time during the epoch. For those wishing for programmatic usage of a MATLAB pupil pre-processing toolbox, the recently published PuPl (Kinley & Levy, 2021) offers both GUI and programmatic solutions, and can also be used in the open-source MATLAB alternative, Octave (Eaton, 2002). Further, it provides the possibility to process epoched or continuous data, and to correct pupil size for gaze position. For Python users, PyTrack (Ghose et al., 2020) and Mathôt and Vilotijević (2022) provide similar functionality, while gazeR (Geller et al., 2020) or pupillometryR (Forbes, 2020) will do the job in R.

### Single trial models

Rather than calculating epoch averages per condition of interest, and running statistics on these group averages, a more recent trend in pupillometry is to model single trial pupil data. While differences in means between populations or conditions form the foundation of psychological research, single-trial analyses – which take variance within subjects into account – can provide insights impossible to observe on the mean level (for a special issue on this topic, see Pernet et al., 2011). For example, one could analyze fluctuations in task performance over trials as a function of pupil diameter, assess the relationship between stimulus and pupil for each trial (see time series methods below), classify the task or state a participant was in during each trial, etc. Importantly, by reporting both within and between subjects and trials variance, a more full picture of the experimental process under consideration can be obtained.

To date in pupillometry research, a variety of single-trial analysis approaches have been used. In some cases, summary statistics like the ones discussed above (e.g., mean pupil size; peak dilation) have been calculated in some time window and entered into a multi-level model, such as a generalized linear mixed model (GLMM). Such approaches allow for nested, hierarchical data and the possibility to model participants, stimuli, participant-by-condition interactions, etc., as random effects. They also allow one to control for co-variates like baseline pupil size and gaze position. However, such an approach still collapses information across time. For a discussion of the limitations of this single-value approach, see Hershman et al. (2022). Possible alternatives include entering time bin as an additional predictor (and calculating the same pupil metric repeatedly in different time windows), or modeling the parameters of the pupillary time course from the data of the full trial. This latter approach has multiple potential instantiations. For example, some have used growth curve analysis (GCA; see e.g., McLaughlin et al., 2022; Wagner et al., 2019; McGarrigle et al., 2017; Geller et al., 2019). Others have used generalized additive mixed model (GAMM; see van Rij et al., 2019 for detailed review and tutorial). And still others have used Bayesian approaches with repeated *t* tests across the time courses of two conditions (see Hershman et al., 2022 for an overview). For further discussion of the influence of time window selection on statistical results, please see Peelle et al. (2021).

Typically, such analyses are focused on differences between conditions, measured via pupil diameter, whether that is in a single-value framework, or with respect to dynamic changes over time. Below, we switch focus to approaches that can be referred to as “signal-to-signal”; that is, analytic techniques that define some relationship between the dynamic pupil signal and a dynamic stimulus of interest (e.g., the amplitude envelope of music or speech). Such approaches are different from the measures shown in Fig. 2 in that they define a relationship between the pupil and some other signal(s), rather than being exclusively based on the pupil signal alone. Please note that these approaches do not represent final statistical models. The output from these signal-to-signal techniques might be chosen to be calculated in a time-binned or single-valued way and entered into any number of final statistical models, based on the researchers’ chosen theoretical framework (e.g., frequentist, Bayesian, linear, non-linear, etc.).

### Correlation

**Fig. 3:**
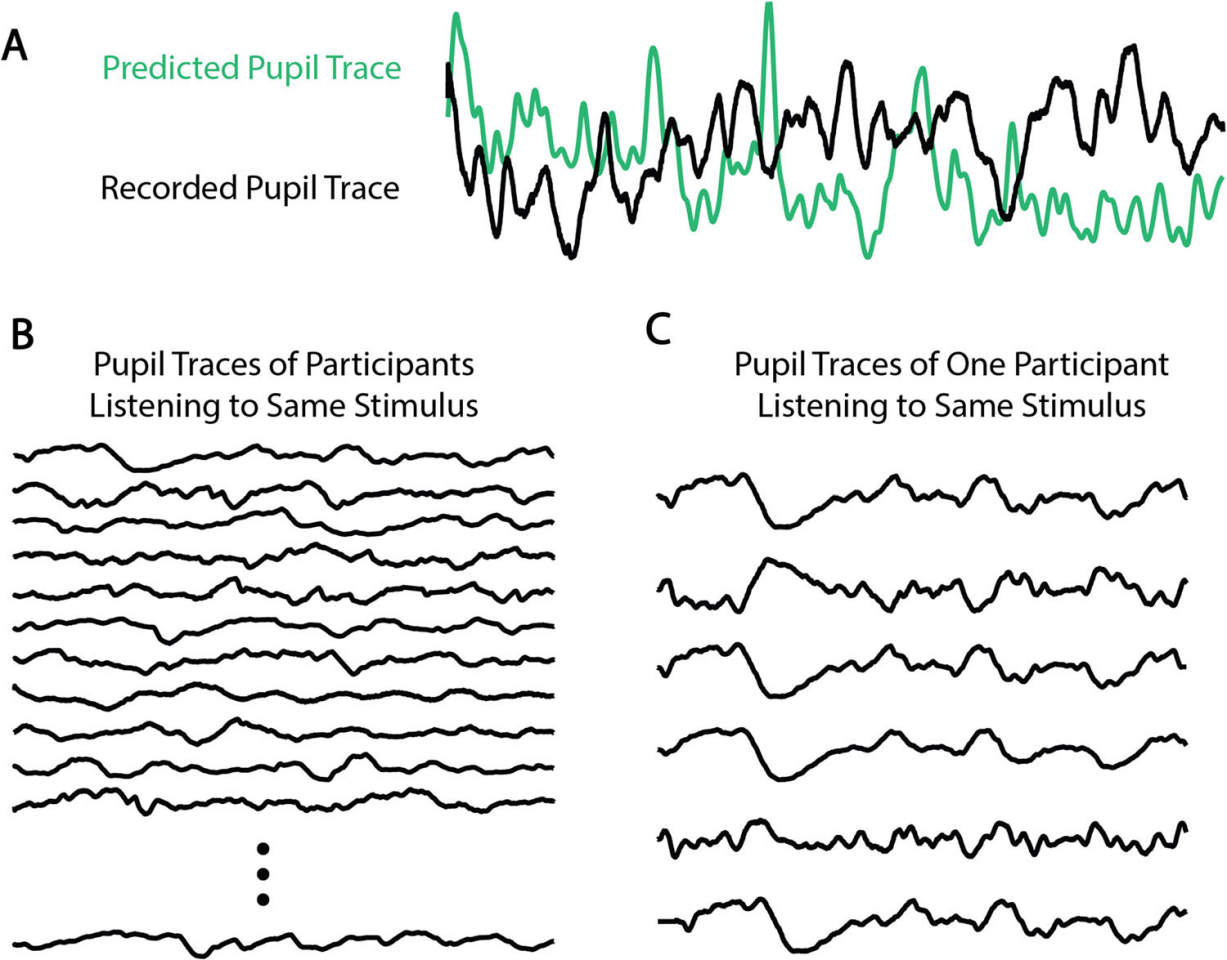
Examples of time series one might wish to compare. **A** Recorded pupil data (black) vs. predicted pupil data (green). **B** Pupil traces of many different participants perceiving the same stimulus. **C** Pupil traces of one participant perceiving the same stimulus. Simulated data; see accompanying code tutorial section *Fig. 3*

Rather than looking at central tendency measures in epoched time windows, there are instances in which one might want to analyze the dynamics of the pupil signal over time. For example, one may wish to compare two or more pupil traces with one another, with a predictive model, or with an attended stimulus (see Fig. 3 for examples), to answer questions such as “Does pupil size change with changes in stimulus feature X?” or “Do participants’ pupil traces synchronize with the stimulus?” The most appropriate analytic technique to answer such questions will depend on the characteristics of the data, as well as the specific mathematical properties underlying the question one wishes to address (see Table 2). It is our goal to provide an overview of the types of signal-to-signal analyses that have been applied in pupillometry and the contexts in which one might wish to use them, so that readers can come to their own informed decisions about what technique to apply to their data. Here, we start with the simple case of computing a correlation, before moving on to more complex methods.

Pearson’s correlation coefficient, which ranges from -1 to 1, is used to index the strength of linear covariance between two times series. The coefficient is calculated as the covariance of the two signals, divided by the product of their standard deviations. The Pearson correlation coefficient is scale-invariant (i.e., *X* or *Y* can be transformed by some constant and the correlation coefficient will not change) and symmetric (i.e., $corr(X, Y) = corr(Y,X)$). While the sign of the correlation coefficient (positive or negative) can be used to understand the relationship of the effects, the square of the correlation coefficient (i.e., the coefficient of determination) is often used as a measure of the proportion of variance one variable can explain in another, ranging between 0 and 1. For example, say one is interested in the correlation between the pupil time series and the amplitude envelope of some audio signal to which a participant was listening. We get a correlation *r* of .6, which we can interpret to mean that when the amplitude envelope of the sound increases so does the pupil size (and vice versa). We can then square this coefficient and say that the amplitude envelope explains $36\%$ of the variance in our pupil time series. Note that, when using Pearson’s product-moment correlation, the two time series to be correlated should be normally distributed and the analysis will only capture a linear relationship between *X* and *Y* (i.e., it cannot be used to analyze nonlinear relations which might exist in the data).

Depending on the properties of the signals (e.g., what stimulus was presented while the pupil trace was recorded, duration of the recording, etc.), it may be that the assumption of stationarity (constant mean and variance over time) is violated. In such a case, one could instead calculate the correlation coefficient over moving time windows (in which the signal could be assumed to be stationary). Such an approach is referred to as a ‘moving,’ ‘rolling,’ or ‘sliding window’ correlation, and yields a time series of correlation coefficients, with which one can then do further analyses.

**Fig. 4:**
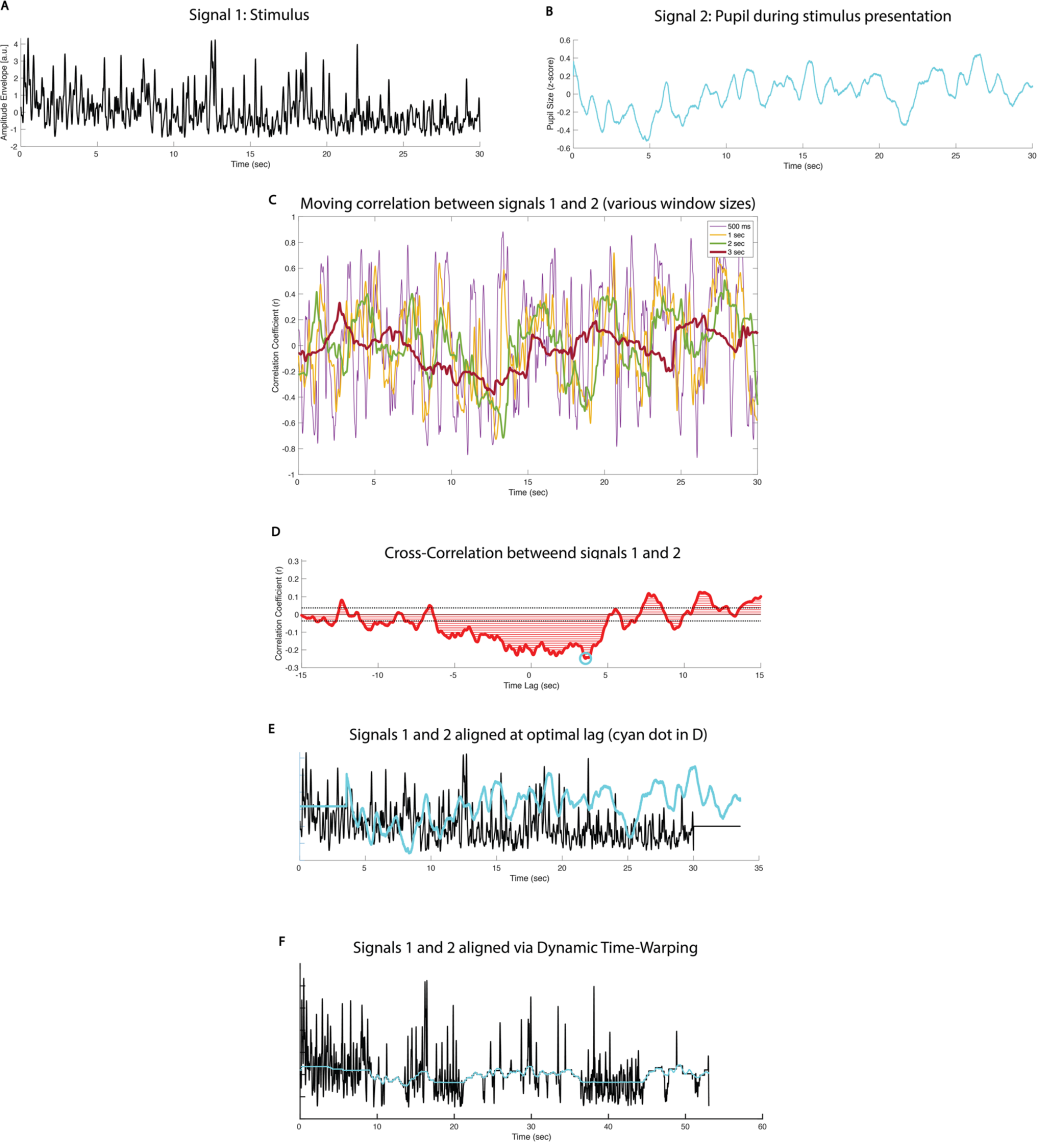
Time-domain techniques for analyzing the similarity/difference between two or more signals. Panels **A** and **B** correspond to two possible signals of interest. Panel **C** shows the moving window correlation between both signals at window sizes of 500 ms, 1, 2, and 3 s. Panel **D** plots the cross-correlation function between the two signals, with their optimal lag highlighted by the cyan dot, while panel **E** visualizes the two signals against one another at their ideal lag (with zero-padding on either side). In this case, the highest correlation (*r* = -.25) occurs when the pupil lags the amplitude envelope by 3.55 s. Instead of using a constant lag, one can allow for variable lag between the two signals by using dynamic time warping (Panel **F**). Here, the distance between the two signals is 2020. Note that the *x* axis of **F** is now extended to 60 s. Please see the in-text sections corresponding to each method for more details. Analysis code to reproduce all examples in this figure is provided in the accompanying code tutorial, section *Fig. 4*

Figure 4, shows two example signals to be correlated (panels A and B). In the current case, the example signals are from the toy data set associated with this paper, which includes the pupil traces of multiple participants listening to the same except of Duke Ellington’s “Take the A Train.” Panel A shows the upper amplitude envelope of this music, while panel B shows the average pupil trace across participants. The Pearson correlation coefficient between both example signals in their entirety is *r* = -.207, *p* < .001. Panel C shows the moving window correlation between the two signals at window sizes of 500 ms, 1, 2, and 3 s. As can easily be visualized, the choice of window size will affect results; the four traces are not always in agreement, with respect to the correlation coefficient at each moment in time. As is also obvious in the plot: the larger the window size, the more smoothed out the variation in correlation coefficient will be. The choice of window size should be made according to what the experimenter deems to be the most relevant temporal scale, given the experiment parameters. Note that, when using a windowed moving correlation approach, if a *p* value for each moment in time is needed, then it is necessary to correct said *p* values for multiple comparisons. Such correction could be accomplished via Monte Carlo simulations or data permutations to find a critical *p* value (though see “Appropriate controls” below about permutation considerations).

When the assumption of normality is violated, Spearman’s rank correlation coefficient can be used to assess the monotonic, but not necessarily linear, relationship between two signals. To calculate Spearman’s correlation coefficient, the two raw signals are first converted to ranks, and then the Pearson correlation of the rank sequences is computed. Due to the conversion of samples to ranks, Spearman’s correlation reduces the effect of outlying data points (e.g., the data point with the highest value will have the highest rank, regardless of the magnitude of the raw value). Spearman’s correlation has also been shown to be more robust for distributions with heavy tails; see de Winter et al. (2016) for simulations and discussion, or Schober et al. (2018) for an accessible tutorial with visualizations. In the toy example in Fig. 4, the Spearman correlation coefficient between both example signals in their entirety is *r* = -.229, *p* < .001. The moving window analyses could also be conducted using Spearman, instead of Pearson, correlation. To re-run these analyses, see accompanying code tutorial section Fig. 4.

Most programming languages have easy-to-install statistics packages which include correlation and cross-correlation (see “Cross-correlation”) functions, including the possibility to select the “type” of correlation to use (e.g., Pearson, Spearman). For example, in Python, one could find such functions in the NumPy, SciPy, or Pandas libraries, while, in R, the stats or tseries packages would be good starting points. The same code recommendations apply for cross-correlation functions (discussed below in “Cross-correlation”).

#### Cross-correlation

Like for correlation, the cross-correlation between two signals can be used to understand the degree to which they change together, however it additionally reveals the correlation at varying temporal lags between the two signals. For example, you might hypothesize a relationship between the amplitude envelope of your stimulus and your pupil data, but you likely do not think the relationship is instantaneous, and may be interested in knowing at which temporal lag your pupil data are most highly correlated with your stimulus.

The cross-correlation is calculated by repeatedly computing the correlation (see “Correlation”) between the two signals at specified lags (i.e., separations in time). The result is typically normalized between -1 and 1, giving a correlation coefficient between the two signals at each temporal lag. One can then, for example, find the lag at which the correlation is highest, to understand something about the temporal delay at which these two signals are most related. In concrete terms, we use our toy data set to show that the highest correlation between the pupil signal and the amplitude envelope of the auditory stimulus being listened to is greatest at a 3.55-s lag (see Fig. 4, Panel D). Other examples include using cross-correlation to determine if pupil size changes are elicited by changes in lens accommodation (Hunter et al., 2000), or by specific neurotransmitter systems (Reimer et al., 2016).

One can also take the cross-correlation of an individual signal with itself (i.e., an autocorrelation) to analyze the degree to which the current vs. past values of the signal are similar to each other. Such an analysis (e.g., of the pupil signal with itself) can reveal whether the signal is random (no peaks in the autocorrelation function) or auto-regressive (decaying correlation coefficients over time lags). Autocorrelation can also be used to find temporal periodicities in the signal (e.g., if the signal tends to show a peak in correlation coefficient every 5 s). Thus, one could even consider comparing features of the autocorrelation function (ACF; e.g., slope, lag of maximum correlation) across different participants’ pupil data or between pupil data and some other stimulus-related ACF of interest. Do note, however, that while analyzing autocorrelation in the pupil signal would be interesting in the context of, for example, music listening, in other contexts, autocorrelation can pose a problem for analyses (e.g., inflating type I error in regression-based analyses). For further discussion of this point, see van Rij et al. (2019).

To look at the correlation between data at time t and time t ± lag without the influence of the intervening samples, a partial autocorrelation can be used. While few pupillometry studies have employed such methodology, a recent paper by Zénon (2017) analyzed the pupil data of five participants passively viewing a rapid presentation of landscape images and showed that all participants’ pupil traces exhibited a shallow, negative ACF slope, and a partial autocorrelation function that converged after about ten lags of 10-Hz samples (i.e., 1 s). Having confirmed the autoregressive nature of pupillary responses, one can then model them using autoregressive models. However, given that Zénon (2017)’s study only involved five participants in a rapid visual presentation paradigm, further research is necessary to determine whether the pupil regularly exhibits autoregressive characteristics in a variety of contexts, across a majority of people, or whether a more structured stimulus (e.g., music) might introduce peaks in the autocorrelation function.

#### Reverse correlation

Reverse correlation aims to estimate unknown variables, for example, a person’s mental representation (Brinkman et al., 2019), or a neuron’s receptive field (Ringach & Shapley, 2004). Reverse correlation is particularly popular in social psychology as a means to unveil the “mental templates” of a participant (for a review and primer on this method, see Brinkman et al., 2017). The basic approach of the method is to present a participant with random variations of stimuli in a two- or four-alternative forced choice task, asking them to judge which stimulus most matches their mental representation of e.g., a woman. The method is referred to as “reverse” correlation because the participant, rather than the experimenter, determines what is “signal” (i.e., relevant) and what is “noise” (i.e., irrelevant).

Typically, reverse correlation has been used with visual stimuli to construct a visual image; however, reverse correlation is also possible in the auditory domain (Ponsot et al., 2018). In fact, it has been used for decades in single-unit neuron studies (De Boer & Kuyper, 1968) to identify the average type of stimulus that elicits an action potential (i.e., a spike-triggered average), as opposed to the traditional method of presenting the same stimulus multiple times and creating a peri-stimulus time histogram of neural activity (for review see Eggermont et al., 1983). Various methodological advancements have further optimized the reverse correlation method; recent work indicates the importance of accounting for shifting sensory weights and decision boundaries to properly estimate and interpret reverse correlation in human studies (Okazawa et al., 2018).

In pupillometry research, Kang and Wheatley (2017) used reverse correlation to relate moments of pupillary synchrony across listeners to the emotional salience of the narrative to which participants were listening. They had a completely separate sample of participants continuously rate the salience of narrative. Then, they reverse correlated moments of high pupillary synchrony (averaged epochs of low dynamic time warping costs; see “Dynamic time-warping” below) with the rated salience values. They predicted that moments of greatest synchrony would correspond to moments of greatest salience ratings, and that is indeed what they found.

Though referred to as reverse correlation, this method is no different mathematically from a standard correlation or cross-correlation. It is rather the conceptual frame that has changed and the approach the experimenter takes to get to a result. One might think of reverse correlation as a “data-driven” or “bottom-up” approach, as it allows the pupil data to show the experimenter what is important in relation to the stimuli presented (as opposed to the experimenter only looking at the pupil data time-locked to events they believe should be of interest).

#### Inter/Intra subject correlation

Rather than comparing pupil time series with stimulus time series, one might wish to compare the pupil signals of multiple participants (inter-subject), or the same participant (intra-subject), with each other. One method for comparing multiple time series at once is inter-subject (or intra-subject) correlation. ISC allows one to identify moments of high correlation across all signals. We will proceed with the inter-subject case. To calculate the ISC, all data for each participant should be in a matrix. A correlation is then calculated across all participants at each time point, using a user-defined sliding window. Fisher’s *r*-to-*z* transformation is applied to the correlation coefficients, then averaged *z* values are inverse transformed back to *r* values.

When interpreting ISCs, note that positive *r* values indicate moments in time where all traces show a consistent change, while *r* values near zero indicate little correspondence between traces at that moment in time. Perhaps a bit counter-intuitive, low (i.e., negative) *r* values indicate moments in time that the traces consistently show a change, albeit in different directions.

If one is additionally interested in which stimulus features lead to consistent responses across participants, the ISC time series can be compared to a stimulus feature of interest using any number of other methods in this section. For example, to identify specific moments in the stimulus that lead to high ISC, one could use reverse correlation (see “Reverse correlation”). Though ISC has only very recently been applied to peripheral physiological measures (Czepiel et al., 2021), including eye movements and pupillometry (Madsen et al., 2021), a multitude of examples exist in the EEG and fMRI literature (Simony et al., 2016; Hasson et al., 2004; Ben-Yakov et al., 2012; Hasson et al., 2010; Wilson et al., 2008; Jääskeläinen et al., 2008). Code to implement ISC can be found in the aforementioned papers or on the Parra lab website (https://www.parralab.org/isc/), though note that this implementation is for EEG Cohen and Parra (2016) and will need to be adapted to single channel pupil data.

### Regression

Regression can be linear or non-linear. The simplest, linear case involves predicting *Y*, given *X*. To keep things concrete, that might be something like predicting pupil size (*Y*), given the amplitude envelope of the stimulus to which someone was listening (*X*). While we used that same example above in the correlation “Correlation” in assessing the degree to which *X* and *Y* changed together, the goal with regression is to fit a line that most minimizes the sum of squared errors between that line and the actual observations. Via this fit, there are some deviations from the mean pupil size, which we can account for, and some for which we cannot. The proportion of error we can account for in relation to the total error is referred to as $R^2$. In “Correlation” above, we discussed the squared correlation coefficient; this value is identical to the ${R^2}$ of a linear regression.

Beyond the most basic form of ordinary least squares linear regression, many more methods exist (e.g., polynomial, lasso, logistic, support vector, Poisson, principal components). Readers should turn to other resources for more detailed mathematical explanations of all these types. In the following, we briefly highlight a special case of regression which may prove particularly fruitful in the domain of pupillometry.

#### Temporal response function

The temporal response function (TRF) has gained recent popularity in EEG and MEG analyses (Lalor et al., 2006; Lalor & Foxe, 2010; Ding & Simon, 2012; Broderick et al., 2018). Theoretically, it is very similar in nature to (de)convolution (discussed above in “Pupillary responsefunction”), in that both aim to understand how an impulse in a particular stimulus feature is mapped onto a physiological response (i.e., one aims to obtain a response function). However, TRFs, as they have been discussed in recent literature, have one important advantage over pure (de)convolution: by using regression and optimizing a cost function, the presence of autocorrelation in the signals (which, as previously discussed in “Cross-correlation”, may exist in pupil data) is not a problem anymore (Crosse et al., 2016). Similar to (de)convoultion, TRF-based analyses can occur in the forwards or backwards direction to (1) predict a physiological response, given the stimulus (forward), or (2) predict (or “reconstruct”) the stimulus, given the physiological data (backward). Depending on one’s direction of interest, regularization techniques to prevent over-fitting may be of more vs. less importance. For example, in the context of EEG data and attended vs. unattended speech stimuli, Wong et al. (2018) have shown that backwards models perform significantly better than forward ones, but rely more heavily on proper regularization. Specifically, forward models can work well with ordinary least squares regression (no regularization), while backwards models do not. In the backward case, Tikhonov regularization (also known as ridge regression) results in the highest accuracies. The segment length over which one attempts decoding also has important theoretical (how often are attention switches likely?) and computational implications and again depends on the context under study, though Wong et al. (2018) suggest an optimal range of 3–5 s (for EEG data).

The canonical correlation analysis (CCA) is an extension of the linear methods for analysis. With CCA, the two signals are projected onto a subspace that maximizes correlation (Thompson, 1984), deriving a set of orthogonal directions in which the two signals are highly correlated. Recently, CCA has been shown to be better than forward and backward TRF models in auditory-EEG analysis by de Cheveigné et al. (2018). Further, deep learning methods also have been explored for improving the canonical correlation between the EEG signals and auditory stimuli, as illustrated by Katthi and Ganapathy (2021).

To date, TRF techniques have not been applied to pupil data by that name, but many papers exist which have used (inverse) general linear models to estimate a response function of the pupil to some type of stimulus (see, for example, Korn and Bach (2016); Korn et al. (2017)). Also, please see “Pupillary response function” above. One possible avenue of future research might include using regression or CCA to decode the relationship between pupillary activity and the amplitude envelope of speech or music (N.B. one could do this for any number of different stimulus features of interest, see e.g., Leahy et al., 2021). Further, by estimating a TRF for each participant individually, one could then compare differences in the parameters of TRFs between participants to determine the degree of pupil response variability for some particular stimulus feature. Such an approach might be particularly useful in characterizing differences in pupillary response functions to certain types of stimuli in clinical populations. Crosse et al. (2016) provide a MATLAB implementation for multivariate TRF analyses; a Python translation can be found here: https://github.com/SRSteinkamp/pymtrf.

### Dynamic time-warping

Because it is plausible that different people may exhibit differing time constants with respect to the relationship between external or cognitive events and pupillary change, and/or that pupil response latency may shift depending on the physiological, psychological, and environmental context of the person, it is reasonable to pursue analytical methods that allow for some flexibility in the time domain when searching for similarities between signals. Dynamic time-warping (DTW) assesses the dissimilarity between two signals by stretching/compressing them to fit each other in a way that most minimizes the sum of Euclidean distances between samples. This process allows for the calculation of a “cost” of warping the two signals to each other (lower cost = greater similarity, higher cost = greater difference between the signals). An example of a DTW result is shown in Fig. 4, panel F. Unlike cross-correlation, which enables discovery of the optimal lag between two signals and the correlation coefficient at that lag, DTW allows lag between the two signals to vary over time (as can be seen by the extended timeline in the figure). In this example, the Euclidian distance between the two signals is 2020 – but it is difficult to interpret such a result. Unlike a correlation coefficient which has a normalized value between -1 and 1, which allows for interpretation of the magnitude of effect without reference to anything else, distances from DTW should be considered relatively (e.g., compare distances between signals 1 & 2 vs. signals 1 & 3 to determine whether signal 1 is more similar to signal 2 or to signal 3).

As with most time series methods, the window of analysis and amount of overlap between sliding windows are important parameters. While only a few studies have employed DTW in pupillometry, the studies to date (in music and language listening domains) suggest a window of 3 s with 1.5-s overlap (Kang & Wheatley, 2015, 2017; Kang & Banaji, 2020). These studies have shown (1) that it can be determined which of two stimuli a participant is attending to, in dichotic listening conditions, by assessing the similarity of the pupil during the dichotic condition to the pupil trace when each stimulus was attended alone (Kang & Wheatley, 2015; 2) that one can predict above chance which of three songs someone was imagining based on their pupil traces recorded while listening to those songs, or, (3) which of four songs someone was imagining, from their previous pupil traces during imagination (Kang & Banaji, 2020). Hence, DTW seems a promising method for future pupillometric studies. For implementation in MATLAB, see accompany code tutorial, section Fig. 4. For R and Python, third-party packages implementing dynamic time-warping are readily available; see Giorgino (2009) and https://dynamictimewarping.github.io/.

**Fig. 5:**
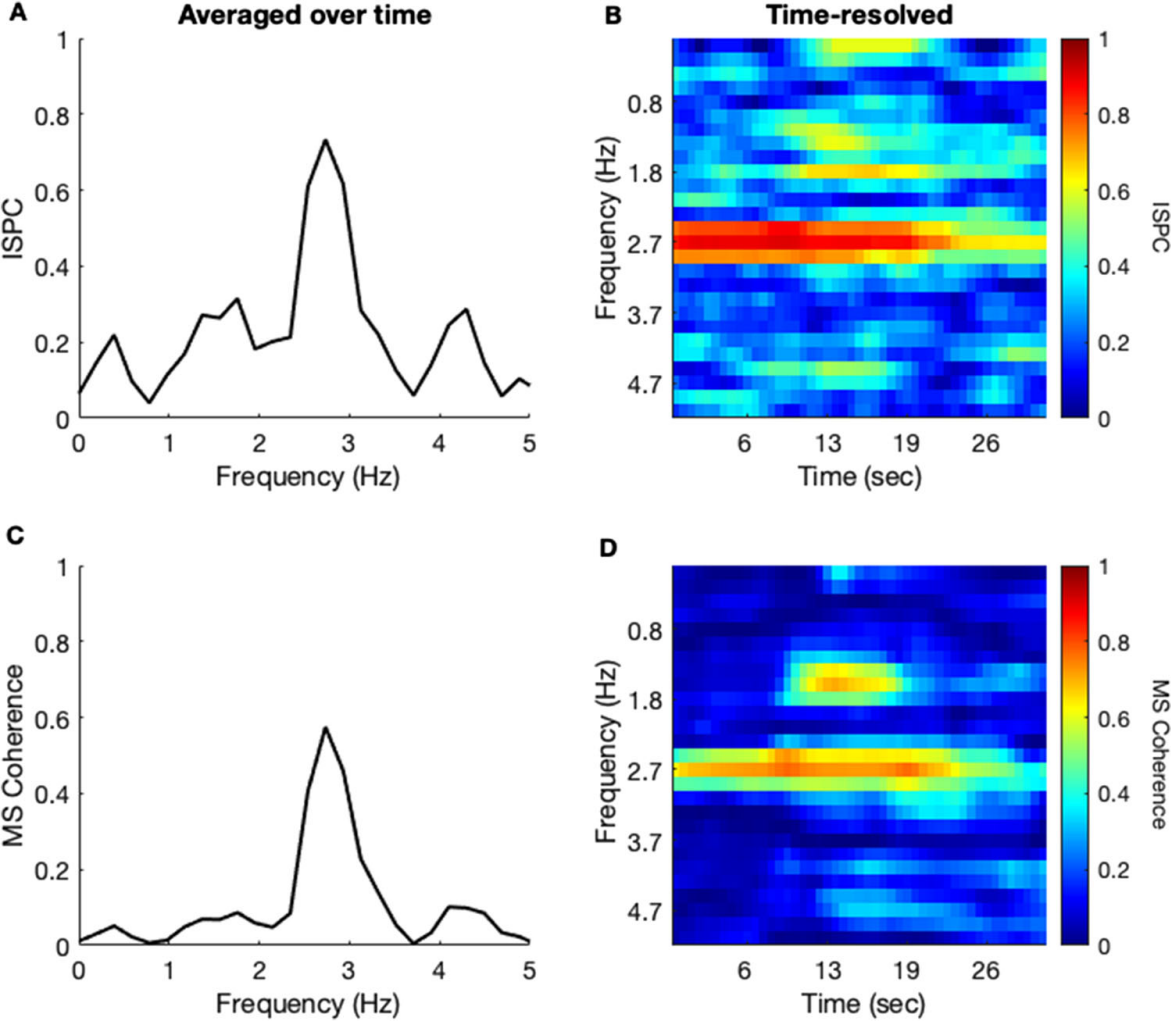
Examples of phase-clustering analyses, between the pupil signal and the amplitude envelope of an acoustic signal. **A** Inter-site phase clustering (ISPC), averaged across time, shows the strength of phase clustering at each frequency. **B** Time-resolved ISPC, for each frequency, at each moment in time. **C**, **D** Magnitude-squared coherence averaged across time and at each moment in time, respectively. Note that magnitude squared coherence takes the power at each frequency into account, while ISPC does not. To recreate this figure, see accompanying code tutorial sections *Time-frequency analyses* and *Fig. 5*

### Phase clustering

One may wish to compare the spectral content of the pupil signal to the spectral profile of the stimulus to determine if they display a consistent phase lag at some frequency of interest, indicating that the pupil has picked up some of the temporal regularities in the stimulus. In such a case, a dependent variable of interest is inter-site phase clustering (ISPC), which measures the synchrony across two (or more) time courses. ISPC is symmetrical (results do not change whether the pupil is factored in relative to the stimulus, or vice-versa), positive, and bounded 0 to 1, with 1 entailing perfect synchrony. ISPC can be used to verify if the distribution of phase angle differences is similarly non-uniform across time points. First, the analytic signal must be obtained for both time-courses, for example using the short-time Fourier transform, and then the vector length corresponding to the difference between phase angles for each frequency point is computed across time points. This is done by first applying Euler’s formula, which outputs the complex polar representation of the phase angle difference for each time point, and then calculating the average vector across time points (Cohen, 2014).

3 $$\begin{aligned} ISPC_f = |n^{-1} \sum \limits _{t=1}^n e^{i(\phi {xt}-\phi {yt})} |\end{aligned}$$

In equation 3, *n* is the number of time points, $\phi {x}$ is the phase angle in radians extracted from the pupil signal, while $\phi {y}$ is the phase angle derived from the stimulus time course at a given frequency *f* and for the same time sample *t*. By calculating ISPC, one highlights which frequency shows high synchrony between pupil and stimulus signals. Note that ISPC is sometimes referred to as “phase-locking value” (see e.g., Assaneo et al., 2021 who use that name for the same equation above). Importantly, equation 3 highlights that information about the amplitude of the signal (or its power) is not considered in the analysis. This means that differences in power do not contribute to synchrony estimation. Furthermore, ISPC is computed over all trial time points. In this regard, if preserving the time dimension in the output is important for the hypothesis one is testing, then it is possible to calculate a time-resolved ISPC using a moving window of sufficient length and overlap to provide a valid profile. Figure 5 highlights an example ISPC analysis of our toy pupil data set. We were interested in the phase consistency between the pupil signal and the excerpt of music people were listening to: Duke Ellington’s “Take the A Train.” Fig. 5A, shows ISPC estimates, averaged across time, between the average pupil time course and the amplitude envelope of the audio signal. As can be seen, there is a clear peak in phase consistency around 2.73 Hz. In fact, this frequency corresponds to what two expert percussionists, as well as two models of musical beat, determined the tempo of the music to be: 163-165 BPM (or $\sim 2.75$ Hz). While this figure shows entrainment of the pupil to the beat frequency, it does not tell us anything about when that entrainment might begin or end. Figure 5B provides an illustration for the time-resolved ISPC of the quite long (30 s) excerpt. ISPC seems to be high for about three quarters of the duration of the music piece, and then it decreases, suggesting a change in tempo or metric clarity. Indeed, when we listen to the audio at that moment in time, some of the instruments drop out and a piano solo begins. The reader should keep in mind that, commonly, trials are much shorter than the 30-s example plotted here (e.g., $1--3$ s). In such cases a reliable estimate of effects would require time-resolved ISPC estimates to be averaged across trials, assuming a sufficient number of trials is available (*N* $> 20$). We refer readers to Cohen (2014) for more detailed tutorials and advice.

### Cross power spectral density & magnitude squared coherence

The cross-power spectral density (CPSD) of two signals is the correlation of two signals in the frequency domain. More precisely, it is the discrete Fourier transform (DFT) of the cross-correlation function of the two signals. As discussed in the correlation and cross-correlation sections above, because a DFT involves the assumption of stationarity, one should be careful in considering whether one’s data meet those assumptions and, if not, to use windows of an appropriate length during which stationarity can reasonably be assumed. Both the length of the window over which the DFT is calculated, as well as the overlap chosen between windows, influence the frequency resolution of the resultant power spectral density estimates. Additionally, the type of window used may introduce artifacts.

Similarly to ISPC, magnitude squared coherence (MSC) reveals the strength of phase relationships between two signals at specific frequencies. The MSC between two signals (*X* and *Y*) is calculated by taking the squared absolute value of the CPSD of *X* and *Y*, and dividing it by the CPSD of *X* and *Y* with themselves:

4 $$\begin{aligned} MSC_{xy} = |\frac{S_{xy} ^2}{S_{xx}S_{yy}} |\end{aligned}$$

where *S* represents cross-power spectral density. Dividing by the auto-spectra normalizes coherence by power, and gives it a value between 0 (independent signals) and 1 (total coherence). However, it is important to note that despite this normalization, coherence results could still be susceptible to bias if phase angles are non-random with respect to changes in power, for example if phase consistency increases but power decreases. Normally, this is not a problem, as phase is independent of power, except when power is very low (Cohen, 2014; Lachaux et al., 1999).

MSC is similar to ISPC, except that power is also included in the calculations. Its inclusion is highlighted in the following equation (Cohen, 2014), which spells out the numerator of the preceding equation:

5 $$\begin{aligned} S_{xy}^2 = |n^{-1} \sum \limits _{t=1}^n |m_{tx} ||m_{ty} |e^{i\phi {txy}} |^2 \end{aligned}$$

In equation 5, the vertically barred *m* of *x* and *m* of *y* correspond to the power of the analytic signals of pupil and input music, respectively, while $\phi {xy}$ is the phase angle difference in radians between the pupil signal and the input music stimulus at a given frequency and for the same time sample *t*. Figure 5C and D plots the magnitude squared coherence averaged over time and across time, respectively, for the same example as in the previous section. Thereby, the difference between ISPC and magnitude squared coherence is made obvious in comparing Fig. 5A and B to C and D.

In the realm of pupillometry, Fink et al. (2018) have used the magnitude squared coherence to show a relationship between a computational model of musical attention and the pupil signal during music listening. They show increased coherence at periodicities predicted by the model, specific to each stimulus, and above chance. In other words, they show pupillary entrainment to auditory rhythms. The observation of a phenomenon such as entrainment requires a method that takes phase into account (i.e., the study of pupillary entrainment is not possible using the traditional methods outlined in “A brief review of epoch-based approaches”).

Beyond analyzing the phase relationship between the pupil and some stimulus, one can also analyze the relationship between the pupil and other physiological signals, for example EEG or heart rate variability, or even neurotransmitters (e.g., in mice, Reimer et al., 2016 show coherence between pupillary activity and both acetylcholine and norepinephrine activity at low and infra-low frequencies, respectively). While functions to compute spectral coherence may not be in the base distribution of popular programming languages, they are readily available in specific packages or toolboxes (Python: csd() and coherence() in SciPy.signal; MATLAB: cpsd() and mscohere() in the Signal Processing Toolbox; R: spectrum() in the stats package. For further discussion and interactive code examples, see accompanying code tutorial sections *Time-frequency analyses* and Fig. 5.

**Fig. 6:**
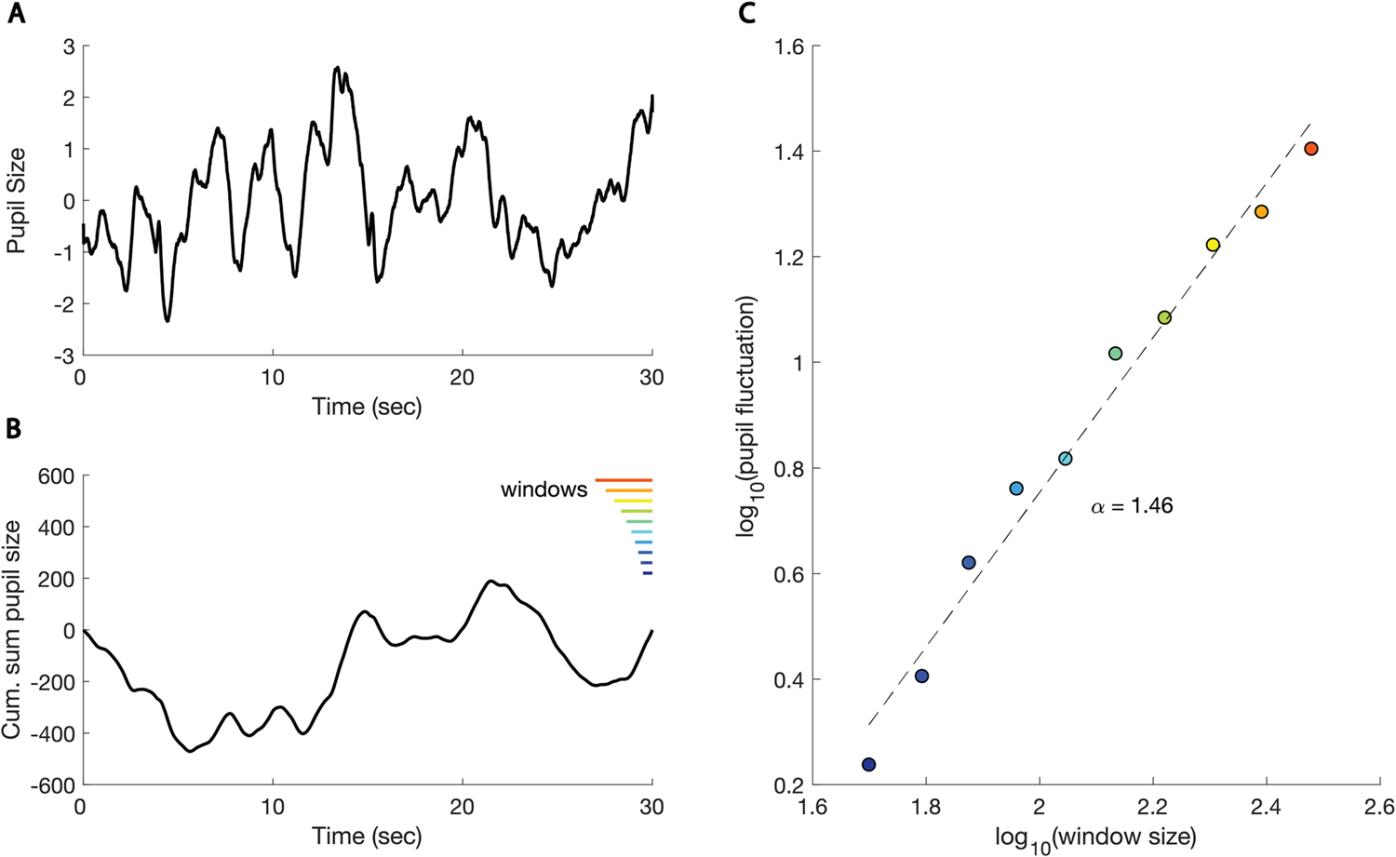
Example of detrended fluctuation analysis (DFA). The *left panels* correspond to pupil data for one participant, one trial (*top*), and the cumulative sum of that pupil data (*bottom*), respectively. In the *bottom left panel*, the *colored lines* indicate different window sizes in which the detrended fluctuation analysis (*right*) was calculated. Note that the window sizes are equally spaced on a logarithmic scale and the largest window size does not exceed $10\%$ of the signal. Here, we used non-overlapping windows when calculating pupil fluctuations in each window, but see discussion about windowing above. In the *right panel*, pupil size fluctuations are plotted for each window length. The slope of the line of best fit connecting these points is referred to as the scaling exponent, or $\alpha$. Values $<.5$ indicate an anti-correlated signal; =.5 an uncorrelated signal (white noise); $.5< \alpha < 1, 1/f$ noise; $> 1$, non-stationary signals, and values approximately 1.5, Brownian noise. Please see “Detrended fluctuation analysis (DFA)” for more details. Code to reproduce this figure is available in accompanying code tutorial section *Fig. 6*

### Detrended fluctuation analysis (DFA)

A number of human behaviors, such as finger tapping, response times (RTs), and memory retrieval have self-similar and scale-free temporal patterns (Kello et al., 2010), meaning they are statistically similar at multiple time scales. Such measures show power-law scaling and long-range temporal correlations (LRTCs), which are a characteristic feature of human behavioral dynamics (Gilden et al., 1995) and brain activity (Linkenkaer-Hansen et al., 2001; Zhigalov et al., 2015). This 1/f type (“scale-free”), arrhythmic activity is distinct from rhythmic (periodic) oscillatory activity (He, 2014), as might be measured via the methods discussed above in “Phase clustering” and “Cross power spectraldensity & magnitude squared coherence”. Indeed, prior research (e.g., Monto et al., 2007) shows that the strength of LRTCs of neuronal oscillations is independent of oscillatory power in a given frequency band. However, it also seems that optimal oscillatory dynamics and sensory processing may in fact rely on the brain operating near what is referred to as a “critical” state (Avramiea et al., 2020). That is, the brain operates near the “critical” point of a phase transition between order and disorder (Chialvo, 2010; Linkenkaer-Hansen et al., 2001; Kello et al., 2010). Operating near criticality is one of the main hypotheses for the presence of LRTCs. It allows for optimal information processing and flexibility in reconfiguration among possible states (Kinouchi & Copelli, 2006; Chialvo, 2010; Avramiea et al., 2020). For example, strong LRTCs were shown to parallel cognitive flexibility, suggesting an advantageous state for task performance (Simola et al., 2017). A recent review (Zimmern, 2020) further demonstrates the clinical relevance of studying brain criticality.

Detrended fluctuation analysis (DFA) (Peng et al., 1994, 1995) is a method for analyzing scaling behavior and it can be used to reveal the presence of LRTCs in a time series (Linkenkaer-Hansen et al., 2001). Hardstone et al. (2012) provide a practical explanation to the DFA algorithm and its underlying theory. To run the DFA, a signal is first normalized to zero mean and then a cumulative sum of the signal is computed. The integrated time series is then segmented into multiple time windows. Within each window, the root-mean-square (RMS) variation is calculated, followed by determination of the typical fluctuation $<F>$ in the given time-scale (i.e., the mean/median of RMS variation of all identically sized windows). In the second stage, the fluctuation for all window sizes is plotted on double logarithmic coordinates to evaluate whether the data reveal power-law scaling. It is therefore important to choose window sizes that are equally spaced logarithmically, so that certain time scales do not have more observations than others. Additionally, it has been recommended that time windows range from at least four samples (enough points for a regression (Peng et al., 1994)) to about $10\%$ of the signal’s length (so that there are enough windows to average over). Overlapping windows may be one solution to increase resolution for window lengths longer than $10\%$ of the signal Hardstone et al. (2012).

The DFA exponent ($\alpha$) is the slope of the trend line in the range of time-scales and can be estimated using linear regression. Whereas DFA exponents $0.5< \alpha < 1$ indicate that there are positive correlations in the time series, $\alpha = 0.5$ indicates that the time series is uncorrelated, and $\alpha < 0.5$ indicates an anti-correlated time series (i.e., fluctuations are smaller in longer time-scales than expected by chance). Values $> 1$ are non-stationary signals, and values of approximately 1.5 are Brownian noise. It is important to report the temporal range over which scaling is observed. In the seminal article by Linkenkaer-Hansen et al. (2001), LRTCs in the amplitude envelopes of ongoing oscillations were analyzed on window sizes ranging from 5 to 300 s. The robustness of LRTCs have later been confirmed also on shorter time-scales in the range about 1-20 s (Linkenkaer-Hansen et al., 2007; Smit et al., 2011). Figure 6 shows an example DFA of pupil for one trial, one participant, so that the reader can get a sense of the basics of the analysis, from raw pupil trace (top left) to cumulative sum (bottom left), to DFA (right) for the windows sizes indicated in the bottom left plot. The DFA exponent could be calculated for every trial / participant and compared between conditions, participants, etc. Note that with larger window sizes, there is greater pupil fluctuation.

The DFA method can reveal how pupil dynamics unfold in time, taking into account different time-scales. It can be applied to pupil size data collected during both resting-state and continuous task performance. It can also be used to quantify and compare pupil dynamics during different tasks or even during presentation of different stimuli. One potential advantage of using DFA is that transient pupillary responses to stimuli are on the order of hundreds of milliseconds or a few seconds and can be ruled out as the source of pupil size modulation on the scale of tens of seconds (see Hardstone et al., 2012). Moreover, computing an average value is often a poor description of scale-free processes, because they typically do not have a characteristic scale. For example, LRTCs in RT time series were uncorrelated with the mean and standard deviation (SD) of RTs (Simola et al., 2017), indicating that DFA taps into different determinants of task performance, otherwise not captured by traditional measures.

The applications of DFA on pupil size data are so far scarce. Onorati et al. (2016) used DFA to show that the pupil exhibits three different ranges of scaling behavior, similar to cardiac dynamics. They also found a higher DFA slope (in the range of 1.5, Brownian noise) when participants recalled autobiographical memories that evoked anger, as compared to sadness or joy. Kaakinen and Simola (2020) used DFA to analyze LRTCs in pupil size time series collected while participants listened to story excerpts and showed that higher story transportation was associated with stronger LRTCs in pupil size fluctuations.

#### Detrended cross-correlation analysis (DCCA)

While we believe DFA, in its own right is an interesting and useful approach for analyzing long-term memory processes in the pupil signal over longer periods of time, the focus of the current paper is on signal-to-signal approaches. So, though one could analyze DFA exponents in condition A vs. B, in keeping with the goals of the paper, we must highlight the signal-to-signal (bivariate) extension of DFA, which is detrended cross-correlation analysis (DCCA). DCCA captures long-range temporal correlations between two (Podobnik & Stanley, 2008), or more (Zebende & da Silva Filho, 2018), non-stationary signals. The resulting detrended correlation coefficient (DCCC) is analogous to the Pearson correlation coefficient (see “Correlation”), but more appropriate for non-stationary time series (Podobnik et al., 2011; Zebende & da Silva Filho, 2018). It is bounded between -1 and 1 and indicates the scale-invariant detrended covariance between two signals. The DCCC can even be calculated online, in real-time, allowing for a range of possible applications in the realm of dynamic physiological data analysis and human-computer interfaces (Kaposzta et al., 2022). Both DFA and DCCA have recently been shown to be robust measures in cases of time series with up to $50\%$ missing data (Zebende et al., 2020), further speaking to their relevance for real physiological signals, like the pupil time course, which may involve data loss for various reasons (see “Discarding trials in which too many pupil data points aremissing or noisy”). Implementations of DFA and DCCA are available for Python (Hardstone et al., 2012; Bianchi, 2020), R (Prass & Pumi, 2020), and MATLAB (Ihlen, 2012); see also accompanying code tutorial section Fig. 6: Detrended Fluctuation Analysis.

**Fig. 7:**
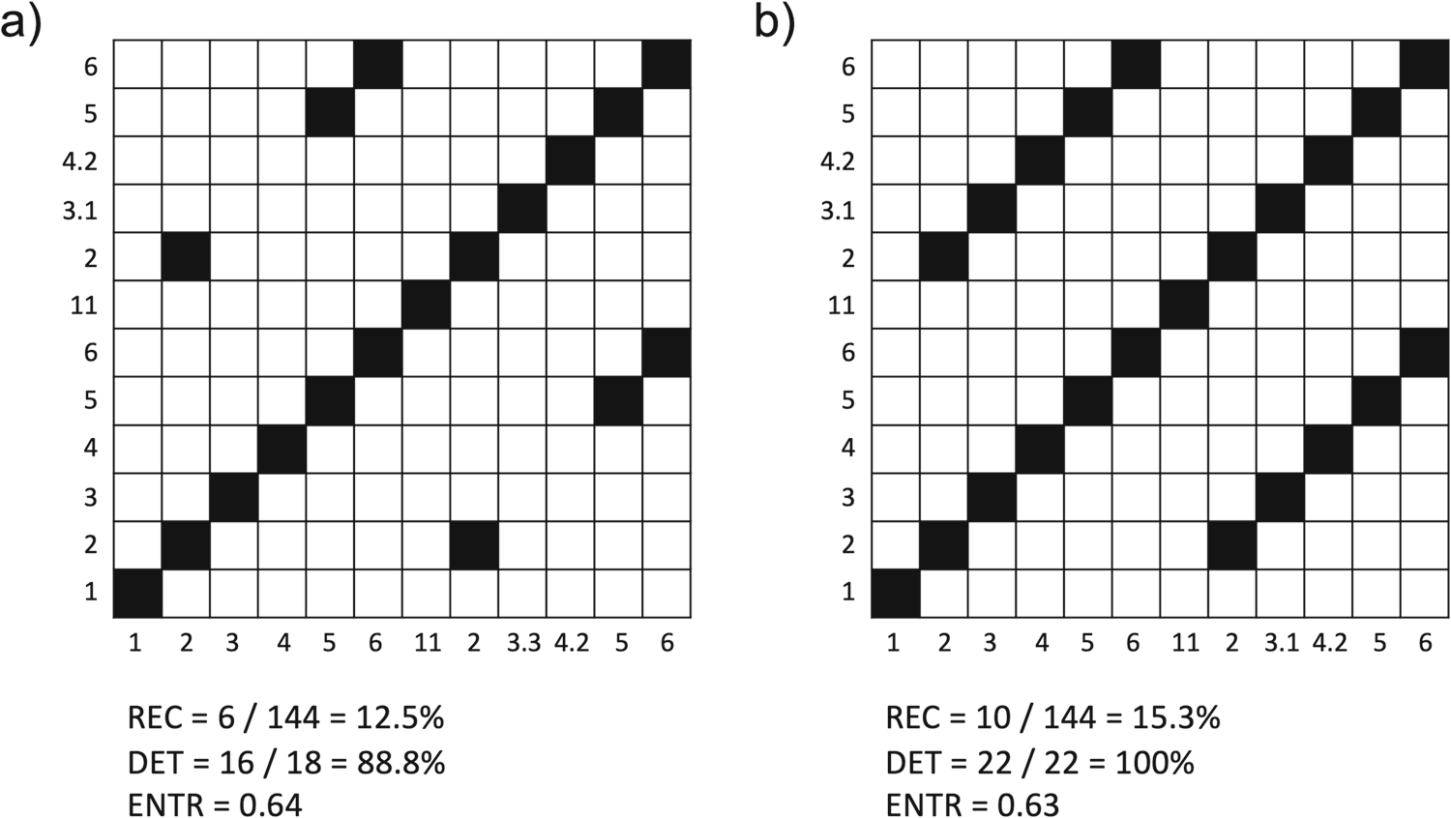
Example RPs. **A** RP with identical recurrences only. **B** RP also including ‘imperfect’ recurrences when a threshold parameter is applied. Below each plot are calculations for three recurrence measures, *REC*, *DET*, and *ENTR*

### Recurrence-based analyses

The previous section introduced a particular kind of auto-correlation pattern, long-range correlations, which capture a type of temporal structure in a time series different from the more classical short-range correlation models, (e.g., auto-regressive models). Recurrence-based analyses offer yet another way to quantify temporal structure, providing a range of auto-correlative measures. There is a wide range of variants of recurrence-bases analyses (Marwan et al., 2007) suitable for different kinds of data and research questions. In the following, we will briefly introduce univariate recurrence quantification analysis (RQA; (Webber & Zbilut, 1994)) which provides measures of temporal structure and complexity for a single time series, such as measures of pupil dilation. Further expansions of this technique exist, for example cross-recurrence quantification analysis (CRQA; (Shockley et al., 2002)), which allows to compare two trajectories – for example two pupil dilation time series, or the co-evolution of a pupil time series with a putative driver signal.

RQA is a versatile method that makes few assumptions and is robust in the face of outlying data points and non-stationarity (Webber & Zbilut, 2005), making it an attractive method to apply to biological signals, such as pupil data. RQA can be used to ask different questions, such as how predictable and stable a time series is, or whether and when qualitative or quantitative changes occur in a time series (Coco et al., 2020). As the name implies, recurrence – that things repeat themselves – is the central concept of RQA. Imagine a simple sequence of numbers such as $x = 1, 2, 3, 4, 5, 6, 11, 2, 3.1, 4.2, 5, 6$. The first six numbers, 1 to 6, are repeated to different degrees of accuracy in the last six numbers. While 1 is not reasonably repeated, 3 and 4 are, say, imperfectly repeated, and the numbers 2, 5, and 6 are perfectly repeated. Now this toy series can be displayed as a so-called recurrence plot (RP).

Figure 7 shows a recurrence plot of the 12 numbers. As we can see, strictly repeated numbers are presented as black squares, while non-recurring numbers are represented as white squares. When we deal with continuously measured data, such as pupil dilation, we will, however, not observe perfect repetition, as such data is subject to measurement error, as well as endogenous fluctuations. Accordingly, one can set a threshold parameter *r*. The bigger *r*, the more tolerance we allow for counting similar, but not identical numbers as recurrence. If, for our current example, we set $r = 0.5$, we include 3 and 3.1, as well as 4 and 4.2 into the category of recurring numbers, while 11 is still excluded (Fig. 7B).

The RP is the basis for quantifying recurrence patterns. RQA provides several measures (Marwan et al., 2007), but the simplest one is percent Recurrence (*REC*), which is simply the sum of all recurrence points (minus the main diagonal) divided by the size of the RP (minus the main diagonal). Other measures can be computed that capture patterning in a time series, for example, how many recurrence points are part of larger repeating sub-sequences within a time series – referred to as percent determinism (*DET*). *DET* is the sum of all recurrence points that have diagonally adjacent neighbors divided by the sum of all recurrence points. Complexity of the time evolution can be captured by the measure *ENTR*, which is calculated as the Shannon Entropy of the diagonal line distribution of an RP (see Fig. 7 for illustrations of *REC*, *DET* and *ENTR*). Note, however, that the recurrence entropy is not equal to entropy of the raw signal, as a signal can be complex, but has comparatively low uncertainty in its temporal evolution. RQA has been applied to eye movement data and pupil dilation data, for example to distinguish between traces of autonomic stimulation in pupil dilation vs. rest (Piu et al., 2019; Mesin et al., 2014), between pupil dilation dynamics of patients with sleep apnea vs. controls (Monaco et al., 2014), or trace effects of affective stimuli (Lanata et al., 2012).

**Fig. 8:**
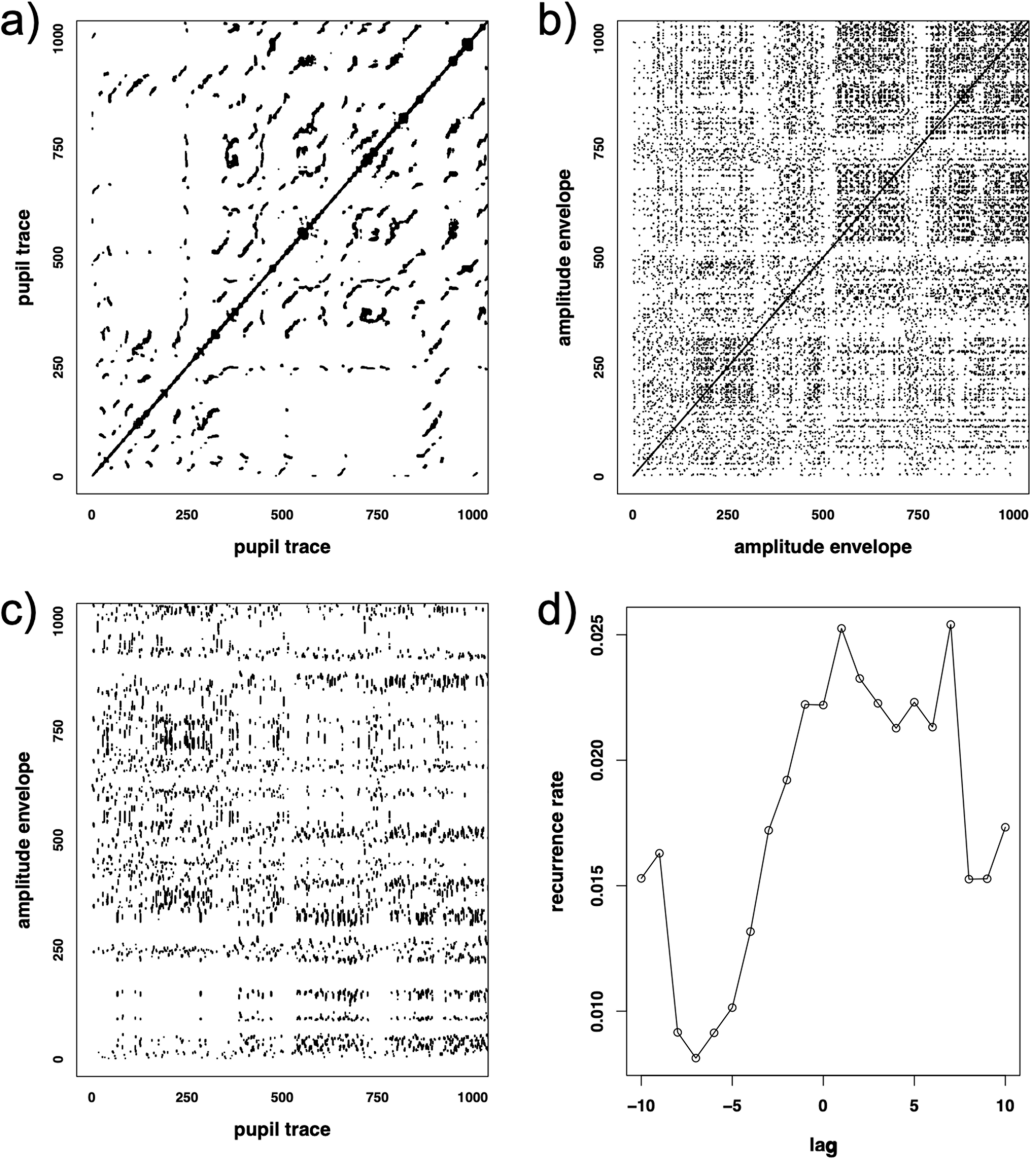
CRQA. **A** RP of pupil trace. **B** RP of amplitude envelope. **C** CRP of pupil trace and amplitude envelope. **D** Diagonal cross-recurrence profile obtained from CRP of pupil trace and amplitude envelope. Higher lagged recurrence means that a signal is following the other signal by a certain lag. The higher overall recurrences point to the fact that the pupil trace follows the amplitude envelope over a range of at about eight lags, with a peak at lag 7. For figure code, see section *Fig. 8* in the accompanying code tutorial

#### Cross-recurrence quantification analysis (CRQA)

Using CRQA, we can also compare two time series. For example, a time series of average pupil trace across multiple participants recorded while listening to an auditory stimulus, charted against the amplitude envelope of that stimulus. Figure 8 shows the individual RPs of the average pupil trace and the amplitude envelope (from Fig. 4A and B), as well as their cross-recurrence plot (CRP). As can be seen from the CRP in Fig. 8C, the central diagonal in the CRP is missing, which means that the two signals are not perfectly time-locked at lag0. Rather, there seem to be cross-recurrences distributed across multiple diagonals.

We can now examine the same RQA outcome variables, *REC*, *DET* and *ENTR* for the CRP. Here, however these measures characterize the average pattern of coupling dynamics between the two signals, unlike in univariate RQA, where they characterize the evolution patterns of a single time series. For the example in Fig. 8C, the values are: $REC = 2.7\%$, $DET = 16.9$, and $ENTR = 0.34$. These values are not always readily interpretable without a proper baseline, control group, or surrogate set (Richardson & Dale, 2005), but overall, coupling seems to be not very complex (low *ENTR*), with comparatively uniform and unique snippets of recurrence of equal size. Furthermore, coupling seems to be mainly due to individual instances of the two series crossing each other, and not much organized in terms of larger trajectories where the pupil trace follows the amplitude envelope over longer periods of time (low *DET*).

If we are interested in knowing more about leader–follower behavior, we can take a closer look at how cross-recurrences are distributed in the off-diagonal, adjacent to the main diagonal (Marwan & Kurths, 2002). This can be done by simply summing up recurrence points in the adjacent diagonals and plotting them as a function of diagonal number, that is, lag. Figure 8D charts the recurrences of the ±10 diagonals off the main diagonal. As can be seen, there is some asymmetry in that there are more recurrences on the right-hand side (the positive lags), meaning that pupil trace recurs more often with amplitude envelope at relative lags between 1 and 8. Hence, the pupil trace is following amplitude envelope, and this following behavior is distributed above the first eight lags, with at peak at lag 7.

In order to conduct RQA or CRQA, further parameters have to be estimated and set; there are also many more measures that are available to quantify the dynamics of a time series (Marwan et al., 2007). The parameter estimation procedure and the description of the different recurrence measures are treated in introductory papers highlighting the analytic approach in R (Wallot & Leonardi, 2018; Wallot, 2017) or MATLAB (Wallot & Grabowski, 2019). A formal introduction is provided by (Marwan et al., 2007). There exist several RQA toolboxes: Norbert Marwan’s CRP Toolbox for Matlab (Marwan, 2017; Marwan et al., 2007) and C (Marwan, 2006; Gordon, 2007), Charles Webbers Toolbox for DOS (Webber, 2021; Webber & Zbilut, 2005), the CRQA-Toolbox in R from Coco and colleagues (Coco & Dale, 2014), and PyRQA toolbox by Rawald for Python (Rawald et al., 2017). Further toolboxes can be found at $www.recurrence-plot.tk$. Code to reproduce the analyses above and Fig. 8 are provided in accompanying code tutorial, section Fig. 8: recurrence quantification analysis.

### Additional considerations

#### Appropriate controls

Note that we have not systematically suggested appropriate statistical controls for each analytical technique. This is because many of the suggestions for each technique are not unique. Specifically, when dealing with time series analyses, there are a few approaches common to all techniques. All of these approaches center around shuffling data or creating surrogate data, but it is the level of data shuffling that needs to be considered carefully. For example, one could shuffle the labels of conditions of interest, or the pupil time series. Shuffling condition, trial, or participant labels is certainly a safe approach, as long as there is a balance between the labels to be shuffled. Shuffling, permuting, or phase scrambling pupil time series data, on the other hand, poses a variety of potential pitfalls.

As has been discussed throughout this paper, pupil signals may display larger-scale temporal dependencies such as auto-correlation or 1/f spectral behavior. Simply scrambling (“randomly permuting”) a pupil time series will give the same distribution of values (mean and variance) in the surrogate time series, but will ruin the spectral properties of the original signal. Comparison to such a surrogate distribution would be to conclude that the true pupil data contains correlated noise or temporal structure. To make more complex comparisons, different types of surrogate distributions should be used. Some possible approaches include amplitude adjusted Fourier transform surrogates or wavelet iterative amplitude adjusted Fourier transform surrogates. However, even these methods may not be appropriate for certain types of synchronization-based analyses, in which case inter-subject surrogates, cyclic phase permutation, twin surrogates, or time-shifted surrogates may be more ideal. We encourage readers to consult Lancaster et al. (2018) for an in-depth review of these, and more, surrogate techniques. The assumed null hypothesis of each surrogate technique, as well as instructions for implementation, are provided.

#### Data sets

To become familiar with the analysis techniques presented above, and to answer new scientific questions which rely on them, researchers need not collect new data. Examples of websites to search for pre-existing, publicly available, pupil data include OpenNeuro.org, the Open Science Framework (osf.io), or Google Dataset Search (https://datasetsearch.research.google.com/). To assist readers in the beginning of this search, we present a non-exhaustive list of 30 pupil data sets: Grenzebach et al. (2021); Bishop et al. (2021); Pajkossy and Racsmány (2019); Kooijman et al. (2021); Winter et al. (2021); Mathôt et al. (2017); Scheepers et al. (2016); Urai (2016); Pelagatti et al. (2020); Lehmann et al. (2019); Chapman and Hallowell (2020); Rozado (2019); Wahn et al. (2016); Nakakoga et al. (2020); Kucewicz (2021); Colizoli et al. (2018b); Moeller et al. (2021); Pavlov et al. (2021); Gee et al. (2017b); Zhao et al. (2020); Lee et al. (2019, 2021); Ribeiro and Castelo-Branco (2021); Clewett et al. (2019); Hanke et al. (2016); Bianco et al. (2021); Madore (2020); Keung (2020); Keitel et al. (2021). These data sets were collected during a range of experimental tasks (e.g., auditory multistability, digit span, bandit task, decision-making, object-tracking, etc.), sometimes in conjunction with other ocular or physiological measures (e.g., photoplethysmography, EEG, intracranial EEG, MRI), in a range of contexts (e.g., across consecutive days, during string quartet performance, while watching the movie Forest Gump, in a clinical environment, etc.).

By using such pre-existing data, the time, money, and energy spent collecting new data sets may be invested in acquiring analytic skills. New questions can be answered from pre-existing data sets. We encourage researchers to make it a habit to first search for pre-existing data sets with which they may be able to answer their research questions and to pre-register their planned analyses related to their research questions before accessing said data sets.

Further, by using multiple data sets to address the same research question(s), larger trends and more robust models may be observable than what could be expected from individual data sets collected in specific contexts and tasks. Of course, there are disadvantages: the researcher does not have control over or access to all variables that may be relevant for the new research question, or the dataset documentation may have gaps which require contacting the original research team, etc. Nonetheless, the potential benefits to the environment, mitigation of human risk, time, and cost are well worth the effort, especially when such effort may afford novel scientific insights.

For those planning to collect new data, we recommend following the suggestions of Kelbsch et al. (2019). Though their suggestions are tailored more so to the study of the pupillary light response, the basic, standardized reporting procedures recommended (recording hardware, participant information, stimulus information, etc.) would benefit all subfields of pupillometry. In the spirit of open science, we urge researchers to release their data (and code) with their research articles, and to see data set creation as an important scientific output in its own right. Such data sets should be organized in a stable and standardized way. To date, there is no agreed upon data file structure for eye-tracking data sets, though the BIDS data format (Gorgolewski et al., 2016), has recently been proposed to be extended to eye-tracking data (see BEP020: https://bids.neuroimaging.io/get_involved.html); however, it is not yet officially implemented. Data set sharing enables reproducibility and future scientific insights unimaginable to the original researcher at the time of data collection. It reduces waste and redundancy, and also allows scientists working in less privileged institutions (where the recording of pupil data may not be possible) to contribute to the advancement of knowledge.

## Discussion

In outlining the psychological and neural underpinnings of changes in pupil size, as well as the variety of innovative ways to pre-process and analyze pupil data, we hoped to introduce researchers to pupillometry, or to reinvigorate their interest in it, and to show the potential application of dynamic, signal-to-signal analysis techniques to a variety of research questions. We have discussed a range of linear and non-linear, temporal and spectral techniques, all of which may prove particularly useful in certain contexts with certain questions. In describing these methods and their example use cases, we aimed at encouraging researchers to choose their analysis technique(s) pre-hoc, that is within a hypothesis-driven approach. A pupil pre-processing and analysis pipeline logically derives from an adequate understanding of the signal being measured (“Introduction”), the potential artifacts present in the signal (“Pre-processing pupil data”), and the type of analysis most applicable to one’s research question (“Analysistechniques”).

For researchers completely new to pupillometry, tools like PuPl (Kinley & Levy, 2021), CHAP (Hershman et al., 2019), GazeR (Geller et al., 2020), and PyTrack (Ghose et al., 2020) provide a starting point for epoched pupil-lometry analyses (see “A brief review of epoch-basedapproaches”). They also bring the research community one step closer to unified pre-processing and analysis pipelines. However, they do not implement all possible pre-processing considerations, nor the signal-to-signal analyses discussed above. Nonetheless, one could use such tools for basic pre-processing and then apply the more complex methods of interest using the software suggestions above and/or custom-written code. We hope that the code tutorial associated with this paper provides a useful starting point for researchers interested in moving towards dynamic time-series-based, signal-to-signal, analysis techniques.

In general, code should not be the barrier to entry for any analyses discussed in this review, as a variety of packages exist for all listed purposes, at least as a starting point, if not an off-the-shelf solution. Thus, it is our opinion that the more pressing and fundamental challenge lies in understanding how each of the pre-processing or analysis techniques one applies transform the data. That is to say, a conceptual understanding is of foremost importance to ensure that an appropriate method is applied in an appropriate way. At times, the default parameters of a built-in function may not be appropriate for a given set of data; it is therefore also critical to read the documentation of the functions being used and choose appropriate parameters (e.g., the order of a filter, the window size of a moving correlation or Fourier transform, etc.).

The scientific insights one can gain are generally limited by the methodological techniques available. While much has already been learned from pupillometry, we believe that methodological advances with respect to both recording equipment and analysis techniques will continue to move the field of pupillometry forward and enable discovery of previously unidentifiable patterns or effects. We hope that the analysis techniques described here will enable researchers to more easily pursue such insights. We believe that the field as a whole will benefit as we move towards shared data, code, and conceptual understanding.

## Data Availability

The toy data set and code required to generate all figures in this paper, plus some additional analyses, examples, and discussion, can be found in the following repository: https://github.com/lkfink/pupilTutorial. An interactive MATLAB script and HTML version of the code tutorial are both provided. For those without access to MATLAB, all code can be run in a publicly available Code Ocean capsule: https://codeocean.com/capsule/1209338/tree/v1.

## Footnotes

1 GitHub: https://github.com/lkfink/pupilTutorial, Code Ocean capsule: https://codeocean.com/capsule/1209338/tree/v1

## Resource Identification Initiative

MATLAB, RRID:SCR_001622

R, RRID:SCR_001905

Python, RRID:SCR_008394
